# Functional mapping and engineering of the Sec translocon unlocked by a cell-free system

**DOI:** 10.1126/sciadv.aej0987

**Published:** 2026-09-02

**Authors:** Markus Meier, Scott A. Scholz, Leo von Bank, João E. Levandoski, Mosche Lückhof, Maximilian Schaaf, Nataliya Safronova, Nathan F. Greenwood, Shutian Si, Ingo Lieberwirth, Anna Lena Jung, Katharina Landfester, Arnold J. M. Driessen, Tobias J. Erb

**Affiliations:** ^1^ Department of Biochemistry & Synthetic Metabolism, Max Planck Institute for Terrestrial Microbiology; Karl-von-Frisch-Str. 10, D-35043 Marburg, Germany.; ^2^ Department of Materials and Bioprocesses Engineering, School of Chemical Engineering, University of Campinas, PO BOX 6066, 13083-852 Campinas, Brazil.; ^3^ Institute for Lung Research, Universities of Giessen and Marburg Lung Center, Philipps-University Marburg, German Center for Lung Research (DZL); Hans-Meerwein-Straße 2, D-35043 Marburg, Germany.; ^4^ Core Facility Flow Cytometry - Bacterial Vesicles, Philipps-University Marburg; Germany.; ^5^ Department Physical Chemistry of Polymers, Max Planck Institute for Polymer Research; Ackermannweg 10, D-55128 Mainz, Germany.; ^6^ Department of Molecular Microbiology, Groningen Biomolecular Sciences and Biotechnology Institute, University of Groningen; Nijenborgh 7, 9747AG Groningen, Netherlands.; ^7^ Center for Synthetic Microbiology (SYNMIKRO), Philipps-University Marburg; Germany.; ^8^ Microbes-for-Climate (M4C) Cluster of Excellence, Synmikro, Marburg.

## Abstract

Almost all membrane proteins are inserted or translocated across membranes by the universally conserved Sec translocon. Despite its central role, experimental access to Sec function has remained limited. Here, we present ProSecCO (Protein Secretion in Cell-free via synthetic Operons), which is a cell-free protein synthesis platform that inserts SecYEG into synthetic vesicles, enabling direct testing of Sec in real-time and high-throughput, circumventing longstanding viability constraints. Screening 300 Sec variants in a single experiment, we consolidate three decades of Sec research, while vastly expanding mutant diversity for structure-function insights. Mapping over 30 functionally critical regions that modulate Sec activity across three orders of magnitude, we uncover dozens of super-active translocation variants and one variant of improved insertion activity. We further leverage ProSecCO to increase membrane protein quality and nanobody export, highlighting the potential of our system for advancing applications in synthetic biology and biotechnology.

## INTRODUCTION

Membrane proteins functionalize the phospholipid bilayer of cells and serve in various essential biological processes, including energy conversion, sensing, transport, light harvesting, motility and membrane stability (*1*). Intrinsically, membrane proteins are largely hydrophobic and tend to aggregate in the cytoplasm. Therefore, cells have evolved molecular machineries to facilitate their insertion into and translocation across the bilayer. The prime example is the Sec translocon (SecYEG in bacteria, SecYEβ in archaea, Sec61 in eukaryotes) (*2*). This heterotrimeric membrane complex consists of one of each subunit SecY, SecE and SecG and is essential to life (*3*). Homologs of the central pore protein SecY are found across all domains of life, and SecY can be even traced back to the last universal common ancestor of all cells (*4*).

SecYEG inserts transmembrane proteins into the bilayer in a co-translational fashion. The translating ribosome is directed to the translocon, which integrates the growing nascent chain into the membrane (*3*, *5*). Beyond insertion, SecYEG also serves as primary protein export system. In bacteria, protein export takes place post-translationally and is powered by the cytoplasmic ATPase SecA (*6*). Hydrophobic alpha-helices in the form of transmembrane domains or N-terminal signal peptides act as recognition signals for SecYEG.

Despite decades of research, both, our understanding of SecYEG and attempts to engineer the pore for improved function – whether for biotechnology (e.g., enhanced protein secretion) or synthetic biology (e.g., synthetic cells) – remains constrained. This difficulty stems from the essential nature of SecYEG and its tendency for toxic ion-leaking mutants, which hampers both in vivo manipulation of the pore, as well as heterologous expression in living cells for in vitro assays (*3*, *7*, *8*). Moreover, in vitro assays on SecYEG still required complicated workflows that restrict both high-throughput and quantitative analysis (*9*).

In vitro cell-free protein synthesis (CFPS) offers a solution to above limitations by removing viability constraints, which has been successfully exploited to study numerous essential proteins (*10*). Critically, CFPS can directly insert membrane proteins into phospholipid bilayers (*11*), which has been leveraged to demonstrate insertion of functional SecYEG into empty membranes (*12*, *13*). However, characterization of SecYEG translocation activity still remained semi-quantitative, relying on labor- and time-intensive methods based on protein substrate protection from proteolytic digestion, SDS-PAGE and fluorescence or [^35^S]methionine detection (*9*, *12*).

To overcome these experimental limitations in studying and engineering the Sec pore, we developed ProSecCO (Protein Secretion in Cell-free via synthetic Operons) - a CFPS-based platform that allows the functional reconstruction of SecYEG in blank-membrane vesicles. Our platform enables evaluation of translocation and insertion activities of several reporters via quantitative high-throughput and real-time kinetic readouts (*14*), revealing insights about Sec and providing a convenient gateway for studying membrane and exported proteins. By directing protein substrates to the membrane, our system reduces aggregation during CFPS, increases translocation and promotes correct insertion orientation, which opens new avenues for studying and engineering of the basic functions of membrane proteins and construction of functionalized membranes from the bottom-up.

## RESULTS AND DISCUSSION

### ProSecCO for the in vitro production and characterization of SecYEG translocons

**Fig. 1.**
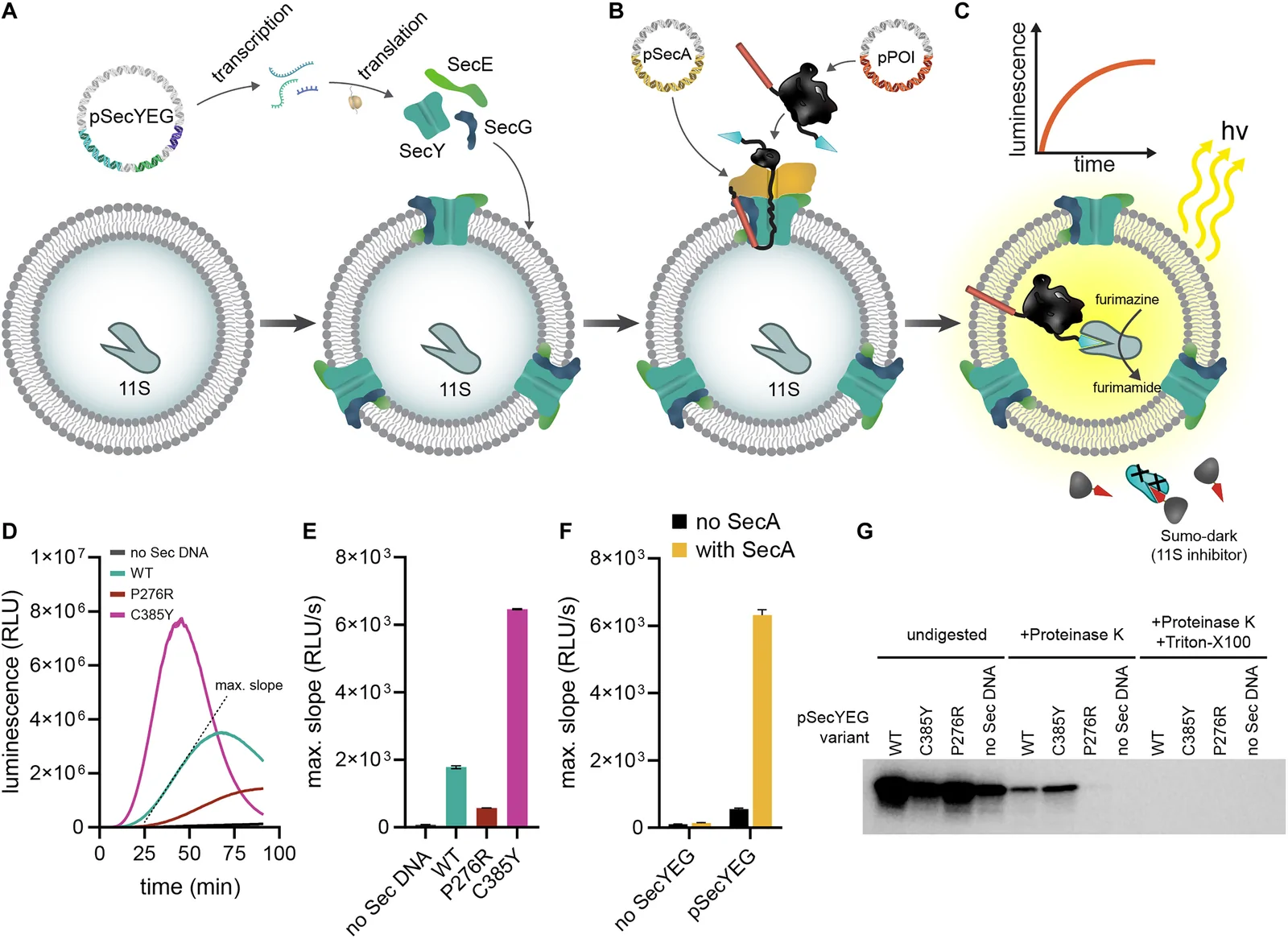
ProSecCO enables the production and characterization of functional SecYEG translocons on blank-membrane vesicles. (**A**) In ProSecCO, *E. coli secYEG* is expressed from a plasmid (pSecYEG) with cell-free protein synthesis (CFPS) leading to spontaneous assembly of functional SecYEG translocons into the blank membrane of reporter vesicles. (**B**) Co-expression of the SecA ATPase facilitates SecYEG-dependent translocation of newly synthesized reporter proteins into the vesicle lumen. (**C**) Schematic representation of the real-time translocation assay based on split nanoluciferase. Successfully translocated reporter proteins with a C-terminal complementation tag activate the encapsulated 11S nanoluciferase fragment leading to a luminescence signal. SUMO-dark proteins block any 11S activity outside of vesicles. (**D**) Real-time translocation data of proOmpA-pep99 reporter in a PURE-based ProSecCO system for SecYEG WT, and SecY variants P276R and C385Y. (**E**) Translocation activity of SecYEG WT, and SecY variants P276R and C385Y, as derived from the maximum (max.) slope luminescence values of (D). (**F**) Translocation activity of SecYEG in the absence and presence of SecA (pre-expressed separately and added during reporter expression). (**G**) Proteinase K protection assay of in vitro translocation activity of SecYEG WT, and SecY variants P276R and C385Y. Shown are luminescence blots of proOmpA-pep86 after transport into vesicles, where they are protected from Proteinase K compared to the control (i.e., vesicles dissolved by Triton-X100). All kinetic data in the figure are shown as mean of technical triplicates, error bars indicate the standard error of the mean (SEM).

To produce the SecYEG pore and quantify its activity in vitro, ProSecCO pairs CFPS of SecYEG in the presence of blank-membrane vesicles with a split nanoluciferase-based translocation assay (*14*). First, SecYEG is produced in vitro from a plasmid (pSecYEG; fig. S2A) and (self-)inserts into the blank membrane of vesicles with an encapsulated catalytically inactive nanoluciferase fragment (11S). Then, translocation activity of SecYEG is probed by production of a plasmid-encoded translocation reporter protein that carries a C-terminal tag for nanoluciferase complementation (*15*). Upon successful translocation of the reporter protein across the vesicle membrane, the C-terminal tag complements the 11S, which results in a real-time luminescence signal from conversion of furimazine into furimamide (Fig. 1, A to C). An 11S inhibitor, SUMO-dark, is provided in the outer solution to prevent any background luminescence from broken vesicles (fig. S1C).

As standard translocation reporter substrate, we employed the first 178 amino acids of the precursor of the *E. coli* outer membrane protein (proOmpA), which is widely used as model for translocation (*16*). We fused proOmpA with a high affinity nanoluciferase complementation tag (pep86) at the C terminus (proOmpA-pep86) and provided the gene on a separate plasmid. For vesicle encapsulation of the 11S nanoluciferase fragment, we chose a molar phospholipid ratio of 40% DOPC, 30% DOPE and 30% DOPG (*17*, *18*). Co-expression of pSecYEG and pSecA, encoding the motor protein SecA, followed by translocation reporter proOmpA-pep86 in a cell-free *E. coli* S50 autolysate resulted in a luminescent signal. In contrast, luminescence was strongly reduced for SecY mutants P276R, G240D and Y429D (*19*–*21*), which are known to be impaired in SecA-based translocation (fig. S3A), confirming basic functionality and sensitivity of ProSecCO.

To further improve ProSecCO, we replaced the high affinity C-terminal tag pep89 with one of two lower affinity tags (pep99 and pep104), which we further call ‘medium-’ and ‘low-affinity’ tag, respectively (*15*). This reduced the overall signal, increasing the assay’s lifetime while opening up the upper dynamic range towards engineering highly active Sec variants (figs. S1, A and B, and S2, E and G). During reporter expression, we followed luminescence for 2 hours and used the maximum slope of the luminescence signal to quantify translocation activity. Compared to SecY WT, the impaired P276R variant of SecY showed ∼5-fold reduction in translocation, while the previously described SecY C385Y mutant with improved translocation activity (*12*) showed a ∼7-fold increase in translocation activity in our *E. coli*-based cell-free in vitro system (fig. S3, C and D).

*E. coli* cell-free extracts still contain all proteins, including Sec components and auxiliary proteins. Thus, we switched to the PURE expression system, a CFPS comprised solely of purified components (*22*), to determine whether the expressed SecYEG and SecA alone were sufficient for translocation. PURE-based ProSecCO recapitulated the results of the *E. coli* system (Fig. 1, D to F), and was independently confirmed through a Proteinase K protection assay (Fig. 1G). Consistent with our cell-free extract experiments, SecY variants P276R and C385Y showed ∼5-fold reduced translocation efficiencies in PURE-based ProSecCO, while the C385Y variant performed ∼4-times better compared to SecY WT (Fig. 1E). However, we noticed that the absolute luminescence signal in PURE-based ProSecCO was an order of magnitude higher than in S50 autolysate, which we traced down to the presence of native membrane vesicles in the autolysate that did not contain the 11S protein, thus lowering the luminescence signal (fig. S5). We used this insight to further tune the dynamic range of ProSecCO in PURE by adding 3 mg/mL blank liposomes without 11S as “competitor vesicles” for all subsequent assays (fig. S2, B and C), establishing a robust quantitative readout for SecYEG translocation through ProSecCO.

### ProSecCO-based analysis of SecYEG-assisted membrane protein insertion

**Fig. 2.**
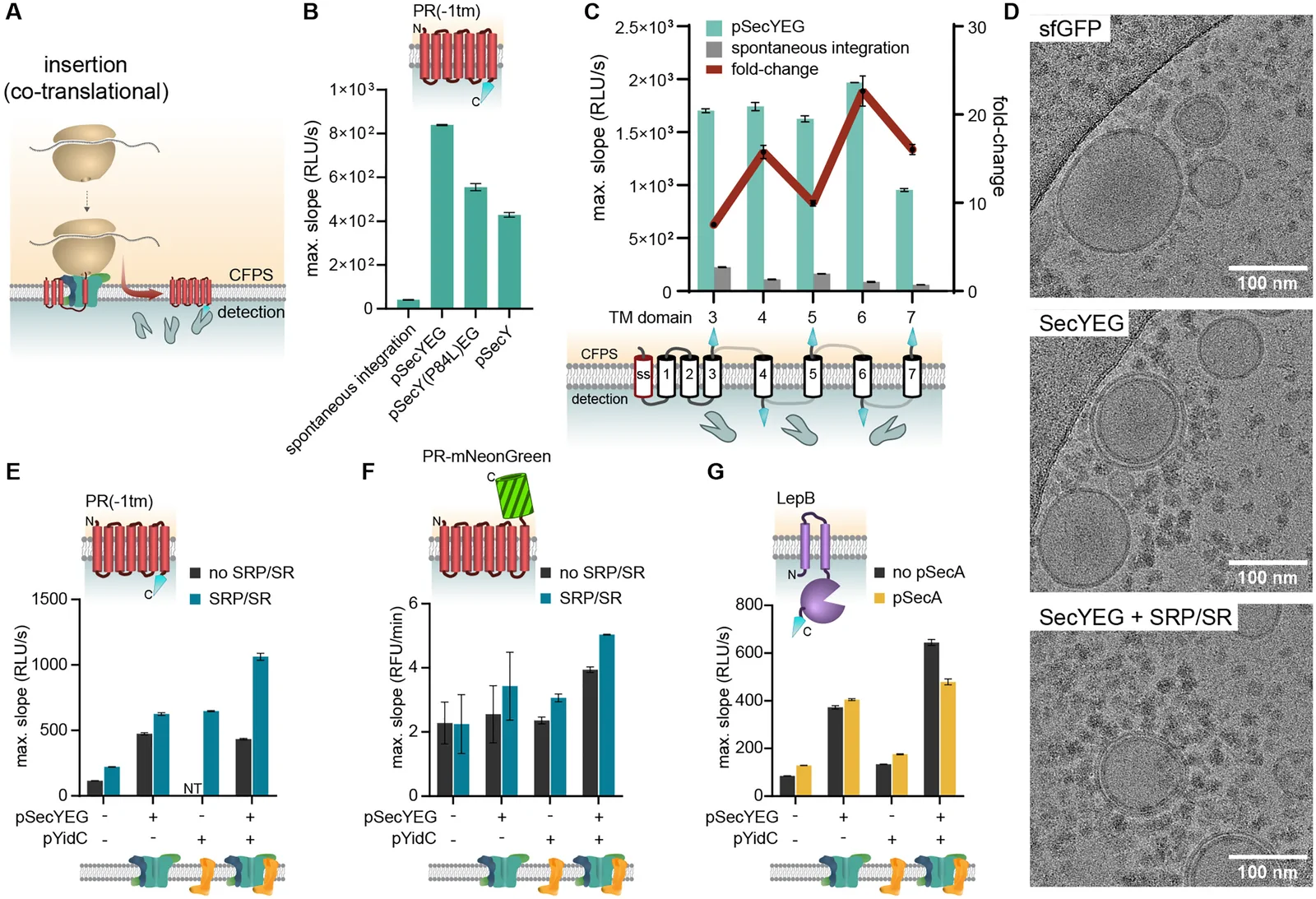
ProSecCO-based analysis of membrane protein insertion via SecYEG. (**A**) Schematic of the luminescence assay measuring co-translational insertion activity of integral membrane protein reporters through SecYEG, using the seven-transmembrane (TM) protein proteorhodopsin (PR) with its C-terminal TM domain replaced by a complementation tag (PR(−1tm)-pep104) as reporter. (**B**) PR(−1tm)-pep104 insertion activity (maximum slope) of pre-expressed SecYEG WT, variant P84L, and SecY alone; “spontaneous integration” was measured by replacing pSecYEG with non-tagged PR to compensate resource competition. (**C**) Directionality of insertion via SecYEG WT, assessed with five PR-based reporters with TMs progressively removed from the C terminus. The red line shows the fold-change in luminescence signal normalized to the “spontaneous integration” control (non-tagged PR replacing SecYEG), following the expected alternating pattern. (**D**) Representative Cryo-TEM micrographs of individual vesicles (∼100 nm diameter) from sfGFP expression, or SecYEG expression without and with SRP/SR; the latter shows more membrane-associated ribosomal particles. (**E**) Influence of auxiliary subunits YidC and SRP/SR on PR(−1tm)-pep104 insertion. pYidC was co-expressed with pSecYEG; SRP (Ffh) and SR (FtsY) were pre-expressed separately and added to the pSecYEG CPFS reaction. (**F**) Influence of auxiliary components on PR insertion, measured by fluorescence of a PR-mNeonGreen fusion reporter. (**G**) Influence of co-expressed pYidC and pSecA on insertion and translocation of the alternative reporter LepB-pep86. Resource competition in (E), (F), and (G) was compensated with expression of sfGFP (luminescence assays) or MBP (fluorescence assay) as negative controls. All kinetic data in the figure are shown as mean of technical triplicates, error bars indicate the SEM.

Having established ProSecCO to track SecYEG translocation activity, we next sought to develop a read-out to measure insertion of integral membrane proteins. As model substrate, we selected the seven transmembrane domain protein proteorhodopsin (PR) that is inserted through SecYEG (*23*). We deleted the last transmembrane domain of PR and fused the low-affinity complementation tag pep104 to its C terminus (PR(−1tm)-pep104), so that upon correct integration of PR, the tag becomes exposed to the vesicle lumen, complementing the 11S fragment, resulting in a luminescent signal (Fig. 2A).

Integration of the PR reporter was dependent on SecYEG, with a 20-fold increased luminescent signal over the spontaneous integration control (i.e., additional expression of a non-tagged PR instead of SecYEG to compensate resource competition), consistent with the central role of the Sec pore in membrane insertion (Fig. 2B). In contrast, SecY variant P84L, which is insertion-impaired (*24*), or SecY alone (without SecE and SecG) showed only ∼50% of WT activity. When neither SecYEG nor non-tagged PR was pre-synthesized, the PR reporter exhibited a noticeable insertion signal corresponding to spontaneous membrane insertion at high expression levels (fig. S6, A and B), which is in line with earlier findings (*11*). However, under these conditions, unassisted insertion of PR resulted in significant aggregate formation and vesicle clumping, which we independently confirmed by dynamic light scattering (DLS) (fig. S7). This aggregation was strongly reduced in the presence of SecYEG, likely due to a larger fraction of correctly inserted PR, demonstrating that SecYEG-assisted insertion drastically improves (transmembrane) protein production quality.

Membrane protein orientation is determined by retention of positively charged loops in the cytoplasm (‘positive inside’ rule) and at least partly controlled by SecY (*25*). To assess how SecY-mediated insertion influences orientation in vitro, we generated five PR-based reporter constructs in which the C-terminal transmembrane helices were progressively deleted, causing the nanoluciferase-complementation tag to alternate between the vesicle interior and exterior. To quantify improvement in directionality through Sec-assisted insertion we plotted the Sec-insertion signal against non-assisted spontaneous integration (Fig. 2C). Surprisingly, we noted that SecYEG seemed to integrate reporters in either direction indicated by generally high fold-changes (Sec-assisted vs. spontaneous integration). Albeit, in presence of SecYEG, we observed a modest, yet clearly alternating luminescence pattern, consistent with modestly improved directional integration of PR by SecYEG (Fig. 2C). Together, these experiments showed that in vitro-produced PR can spontaneously integrate at a basal level, whereas SecYEG drastically enhances overall insertion (∼20-fold), moderately improves directionality (∼twofold) and reduces aggregation in ProSecCo, providing a quantitative readout for SecYEG-based insertion activity.

### Auxiliary factors improve membrane protein insertion via SecYEG

Next, we used ProSecCO to test the impact of auxiliary factors, which are known to enhance and/or aid SecYEG-dependent insertion, particularly in the case of more complex substrates (*3*). We chose three known auxiliary factors of SecYEG, which we co-expressed or provided as pre-expressed protein: SecA, YidC, and the signal recognition particle (SRP)/SRP receptor (SR) pathway. YidC is a membrane protein insertase that inserts integral membrane proteins individually or in concert with SecYEG (*26*). The SRP/SR pathway facilitates sorting of co-translationally integrated proteins to both SecYEG and YidC and consists of the two protein components Ffh and FtsY, as well as the 4.5S RNA (*5*). Because the 4.5S RNA is already part of the CFPS tRNA mix (*27*, *28*), we did not provide or express additional RNA.

When SRP/SR was included in the reaction mixture, PR(−1tm)-pep104 integration was improved by 30% (Fig. 2E), which was likely accompanied by increased association of ribosomal particles with membranes (Fig. 2D). Moreover, SecYEG and YidC showed a synergistic behavior, but only in the presence of SRP/SR, resulting in a more than twofold increased integration over SecYEG alone. This observation supports the finding that SecYEG and YidC form an insertase complex (*29*), which resembles the eukaryotic ‘multipass translocon’ and is independent of the lateral gate (*30*, *31*). To confirm production and correct folding of PR, we used a well-established mNeonGreen C-terminal fusion reporter (PR-mNeonGreen) (*32*, *33*), which independently confirmed that YidC and SRP/SR enhance SecYEG-based PR insertion (Fig. 2F).

We also tested the alternative reporter protein LepB (LepB-pep86), a signal peptidase, which has been proposed to require SecYEG, SecA, as well as YidC for efficient insertion based on temperature-sensitive mutants and crosslinking experiments (*34*, *35*). Notably, LepB insertion was ∼70% increased when YidC was co-expressed, while the motor protein SecA alone, or in combination with YidC, did not further increase the signal (Fig. 2G). Addition of pre-expressed SecA also did not improve LepB insertion, indicating that (at least in vitro) SecA is not essential for LepB insertion (fig. S4B). In summary, these data suggested that – compared to state-of-the art that currently relies on laborious in vivo experiments – ProSecCO is capable of directly quantifying the contribution of different auxiliary factors of SecYEG.

### ProSecCO-based high-throughput screen of a SecY variant library

**Fig. 3.**
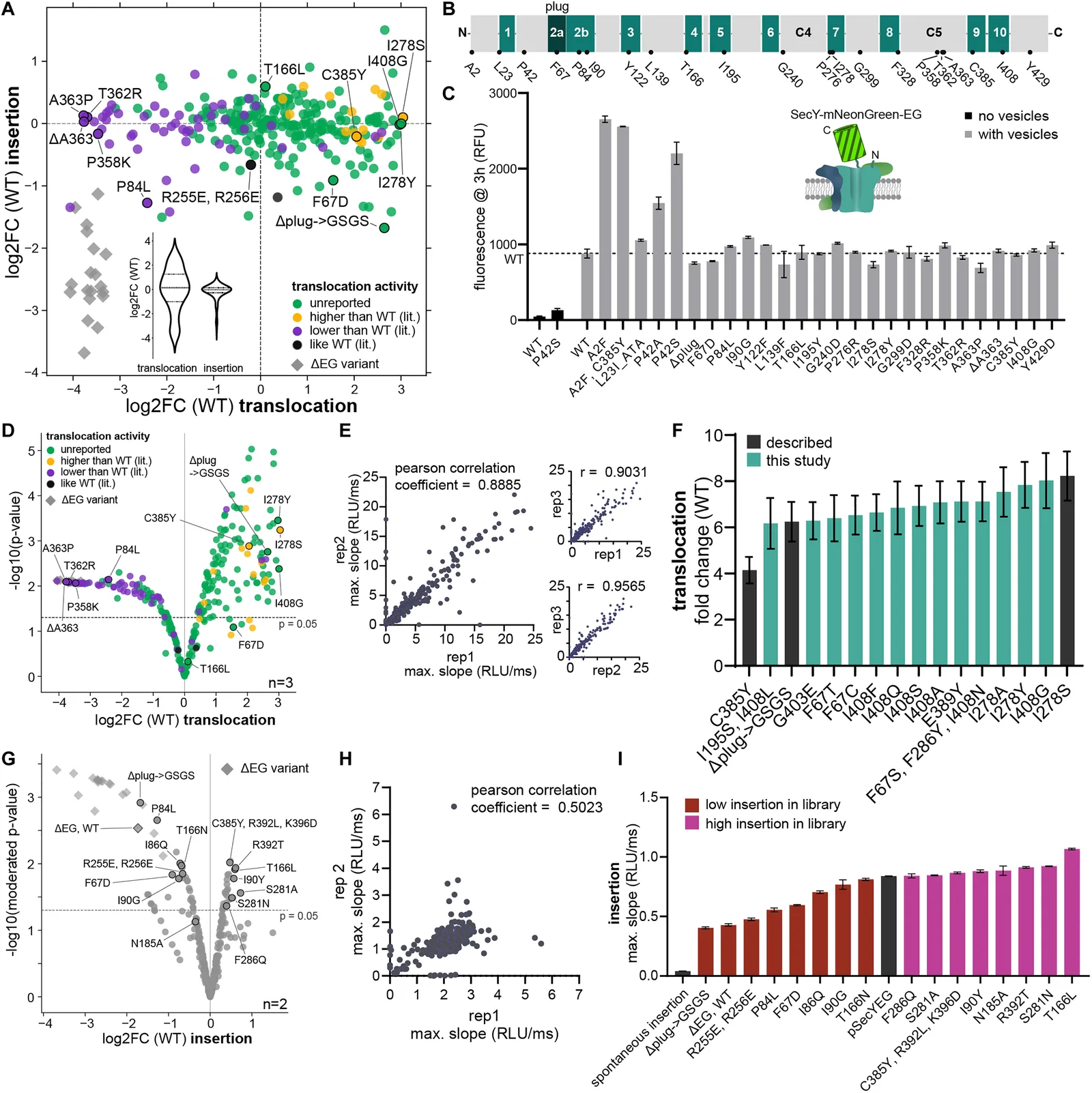
ProSecCO-based screen of translocation and insertion activities of 294 SecY variants. (**A**) Insertion activities of 294 SecY variants plotted against translocation activities, both expressed as log2-fold-change (log2FC) relative to SecY WT (dashed lines); insertion was assessed with PR(−1tm)-pep104, translocation with proOmpA-pep99 plus SecA. Previously reported variants with translocation above or below WT are yellow or purple dots, respectively; new variants from this study are green dots, and variants assayed without SecE and SecG are grey diamonds. (**B**) Expression and folding analysis of 27 selected SecY variants spanning the full protein length, re-cloned with a C-terminal mNeonGreen reporter; transmembrane helices (green), plug (dark green), and SecA interaction domains C4/C5 are highlighted. (**C**) Cell-free expression data of the 27 constructs from linear DNA templates (technical triplicates). (**D**) Volcano plot of translocation activity vs. SecY WT (Welch’s t test, n = 3; color scheme as in (A); dashed line at *P* = 0.05. (**E**) Correlation between replicates of three separate translocation experiments. (**F**) Top 5% SecY variants, showing four- to eightfold improved translocation over WT. (**G**) Volcano plot of insertion activity vs. SecY WT; SecYEG-operon variants are spheres, SecY-alone variants (“ΔEG”) are diamonds, and variants selected for re-analysis are labelled. Given the low replicate number (n = 2) and high replicate variance, data were analyzed with limma (Bayes moderated t test); dashed line at moderated *P* = 0.05. (**H**) Maximum slope values for both insertion assay replicates plotted against each other. (**I**) Re-analysis of the seventeen most striking insertion variants; plasmids were re-transformed, purified, and assayed by manually pipetted insertion (20 μl volume, technical triplicates). All kinetic data in the figure are shown as mean, error bars indicate the SEM.

We reasoned that ProSecCO could serve as a platform for parallel, high-throughput analysis of SecYEG translocation and insertion activities. Therefore, we established an automated contact-free liquid handling workflow (fig. S8A), which we used in the following to re-measure 70 previously described SecY variants, while characterizing 204 new SecY variants within the pSecYEG plasmid, and 20 mutants lacking *secE* and *secG*, all in one single experiment (data S1). The 204 novel variants were deliberately designed to probe mechanistically critical domains of SecY (i.e. pore ring, plug, lateral gate, ribosome and/or SecA interaction sites), highly conserved residues (i.e., P266, I278), atypical hydrophilic residues that face the surrounding lipid bylayer, untypically positively charged residues located at the periplasmic side, as well as previously uncharacterized mutations at described functional sites. The 294 pSecYEG variants tested varied about 140-fold (138-fold, standard deviation 3.5-fold) in their translocation activity. In contrast, insertion activity varied only about 3-fold (standard deviation 1.4-fold), the only exception were the few variants tested without SecE and SecG that were more than 10-fold decreased in insertion activity (Fig. 2A). For translocation, data reproducibility with ProSecCO was very high with Pearson’s correlation coefficients between replicates ranging from 0.889 to 0.957 (Fig. 3, D and E), while insertion activities showed a lower correlation (r = 0.502), consistent with a higher signal to noise ratio observed in our insertion assays (Fig. 3H).

To test for potential biases, we assessed the expression and folding of a subset of 27 SecY variants distributed over the whole length of the protein (Fig. 3, B and C). We used a well-established mNeonGreen reporter (*32*, *33*), which we fused to the C terminus of SecY. Overall, the 27 variants displayed highly uniform expression and folding, with exception of four mutants very close to the N terminus (residues A2 and P42), which showed about twofold increased expression independent of the presence of vesicles. We concluded that the N terminus of SecY was more sensitive towards substitutions, e.g. affecting translation initiation or rate of protein production, and therefore excluded these four points from further analysis.

We first validated our kinetic translocation data against 70 previously reported SecYEG variants. Notably, SecYEG activities reported in the literature are largely qualitative in nature and substantially vary in experimental design and methodology, including reporters and signal sequences used. Despite this heterogeneity in the literature, ProSecCO recapitulated 87% (i.e., 61 out of 70) of the published findings with respect to overall trends, namely increased or decreased translocation activity (Fig. 3, A and D; data S3). In cases of deviations, we could trace four samples back to high variance of individual replicates in our assays. In three cases, we noted a difference between our results and the literature: variants that had been reported to exhibit reduced translocation appeared to be more active in our assays, which is consistent with the position of these mutations in hotspots for super-active variants. In summary, this analysis confirmed the general feasibility of ProSecCO as quantitative screen for translocation activities of SecYEG variants.

**Fig. 4.**
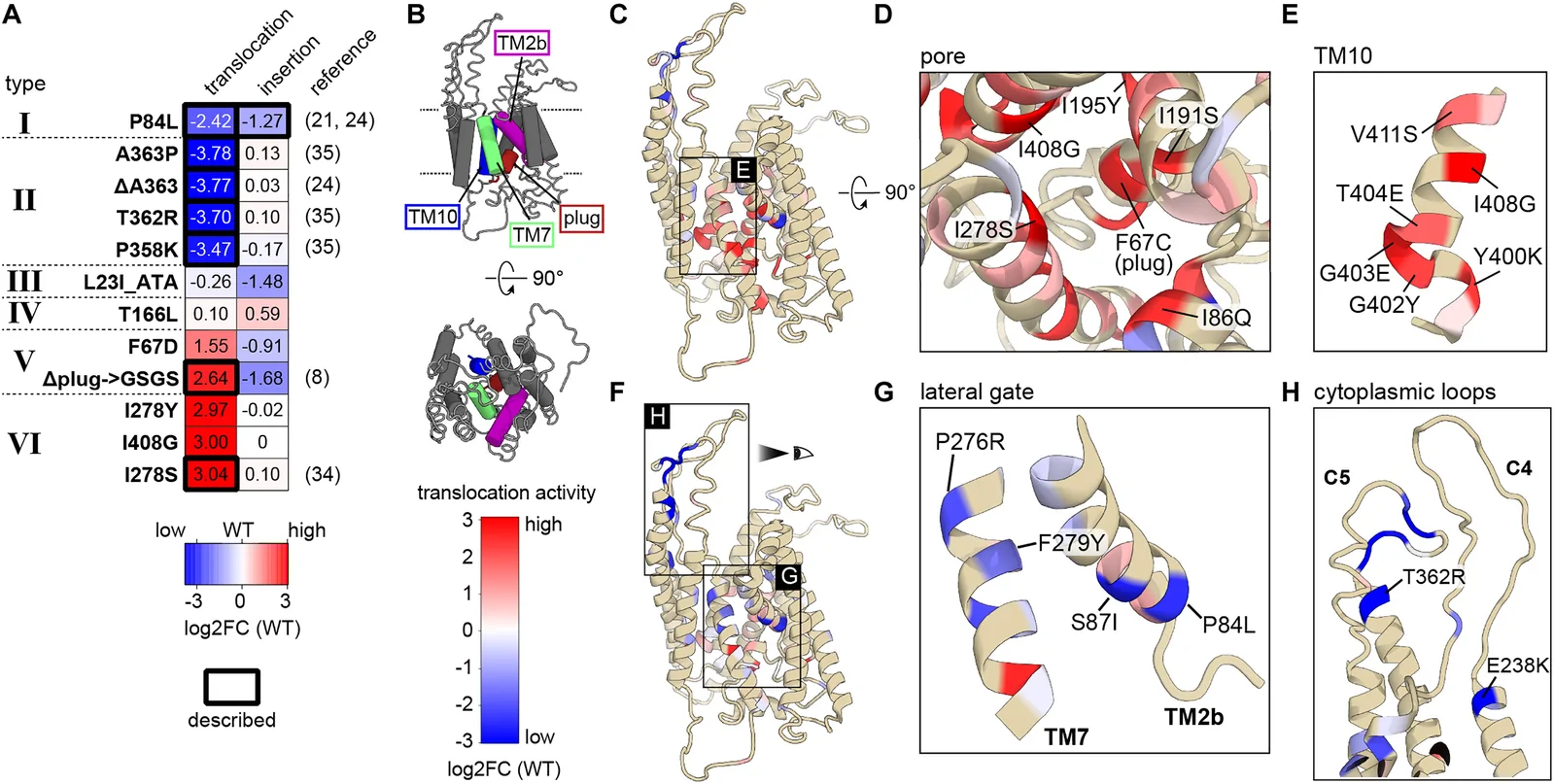
Functional and structural insights derived from the SecY library. (**A**) Heatmap of selected SecY variants and their individual translocation and insertion activities measured via ProSecCO. Variants that were previously described in the literature are boxed in bold. SecY variants were classified according to their translocation/insertion activities relative to WT SecY: (I) low/low, (II) low/WT, (III) WT/low, (IV) WT/high, (V) high/low and (VI) high/WT. (**B**) Schematic representation of the SecY structure. Highlighted are TM2b (pink), plug (*8*) (red), TM7 (green) and TM10 (blue). (**C**) Point mutants with the highest translocation activity obtained for each tested residue plotted on the SecY structure according to their log2-fold-change over the WT activity. (**D**) View from the cytoplasmic side into the central pore of SecY with important mutants of pore ring residues I86, I191, I278, and I408, as well as plug residue F67 highlighted. (**E**) Zoom into TM10 that displays a high propensity for super-active translocation mutants. (**F**) Point mutants with the lowest translocation activity obtained for each probed residue. (**G**) Zoom into the lateral gate helices TM7 and TM2b. (**H**) Zoom into cytoplasmic loops C4 and C5, responsible for ribosome and SecA binding. All values were plotted on a previously published structure (*23*) (PDB: 5ABB).

We next analyzed the full dataset in respect to altered translocation activity. Beyond confirming C385Y as highly-active translocation variant (*12*), we identified 77 additional SecY variants across 39 distinct sites with at least twofold and up to eightfold improved translocation activity, including I278S (*36*) and novel variants I278Y, I278A, E389Y, G403E, I408G and I408A (Fig. 3F). Thus, ProSecCO allowed us to consolidate three decades of SecYEG research and to directly compare previously reported and newly identified SecY variants within a single experimental setup side-by-side (Fig. 4A; data S1). To elucidate the sequence-function relationship of translocation mutants, we mapped our results onto a structure of SecY (PDB: 5ABB). Mutants in the C4 and C5 loops led to severe translocation defects (Fig. 4, F and H), which is in line with the role of these sites as major contact points for SecA and the ribosome (*37*–*39*). Conversely, several known sites [F67 (*40*), I195 (*41*), I278 (*36*), G403 (*24*) and I408 (*41*, *42*)], and new positions (F64, L72, M83, I86, L285 and G402) within the transmembrane regions of SecY gave rise to super-active mutants, and identified TM10 as major hotspot for improved translocation activity (Fig. 4E; fig. S8). Especially striking was the high frequency of super-active translocators generated by diverse substitutions at the hydrophobic pore ring of SecY, which conveys proof-reading of translocated substrates via conserved isoleucines (*43*) (Fig. 4D).

We also analyzed the SecYEG library in respect to integration of PR(−1tm)-pep104 (in the absence of SecA). In contrast to the strong effects observed for translocation, most SecY variants showed only little variation in insertion activity (see above). Thus, we selected the seventeen most striking candidates for an in-depth analysis (Fig. 3G). This analysis confirmed rather modest impact of SecY mutations on insertion activity, emphasizing the need for follow-up experiments in case of high-throughput insertion screens (Fig. 3I). Interestingly, translocation and insertion activities in our SecY variants did not show any correlation, supporting that translocation and insertion pathways are under (partially) different constraints (Fig. 4A; fig. S9, A and B). Notably, we identified one variant, T166L that showed ∼30% increased insertion activity at WT-like translocation (Figs. 3I and 4A). Interestingly, T166 is an unusual polar amino acid within the transmembrane region of TM4 that is located just beneath the hydrophilic groove of TM3–4, a region that was recently described to exert chaperone activity during insertion (*44*). In summary, our data highlighted the potential of ProSecCO to derive a comprehensive, quantitative and directly comparable overview of the translocation and insertion activities of hundreds of SecYEG variants, confirming earlier findings for published variants and identifying novel Sec pore variants with altered translocation and/or insertion activities.

### ProSecCO-based optimization of signal peptides for nanobody translocation

**Fig. 5.**
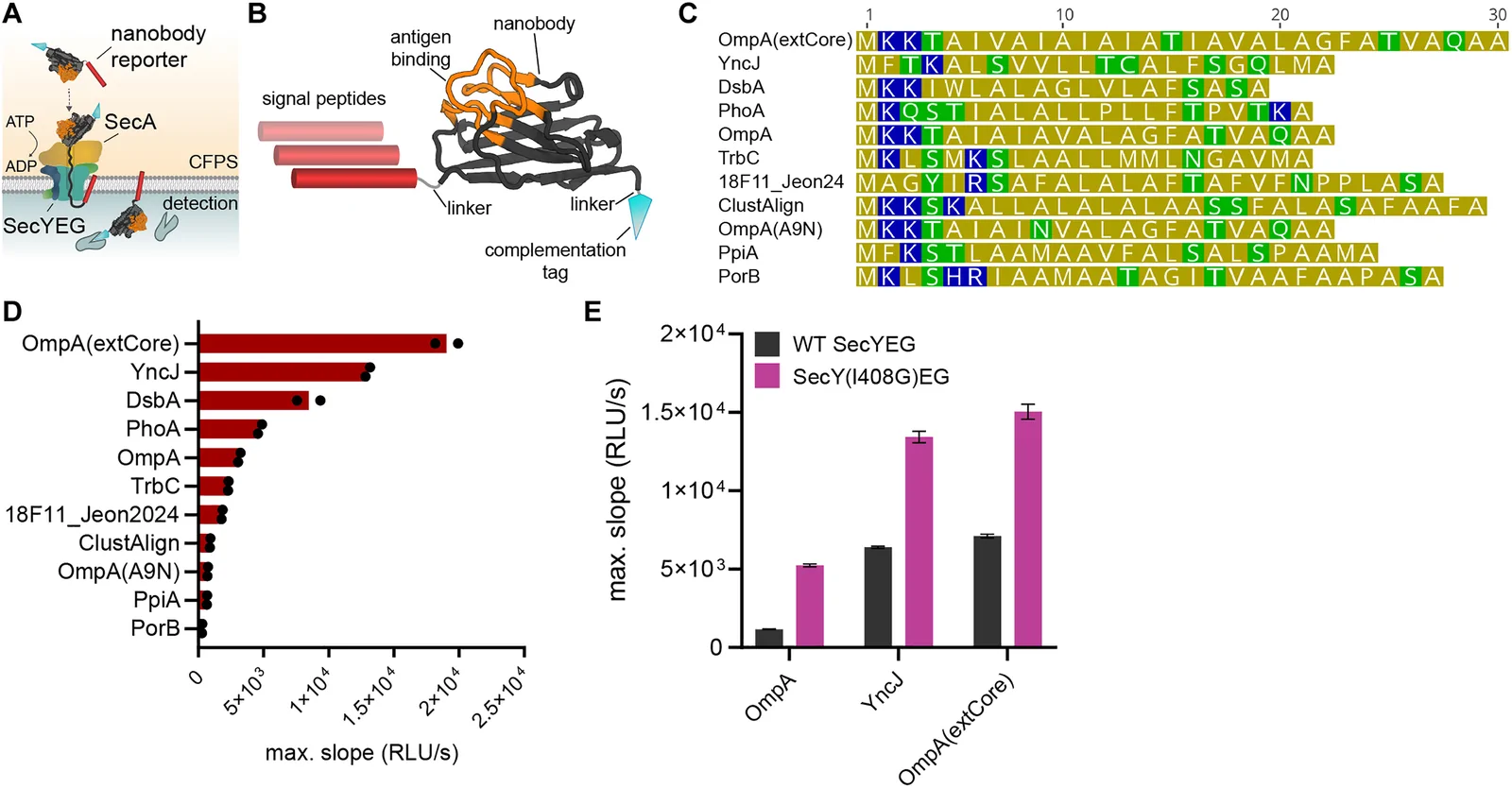
ProSecCO-based optimization of signal peptides and SecY pore for enhanced nanobody translocation. (**A**) Schematic representation of the luminescence assay for measuring SecYEG- and SecA-dependent nanobody translocation. (**B**) Structural prediction of the anti-mouse-IgG nanobody TP1170 reporter with an N-terminal signal peptide and a C-terminal complementation tag. The nanobody structure was predicted with AlphaFold. (**C**) Overview of the 11 different amino acid sequences that were tested as N-terminal signal peptides. Amino acids are provided as one-letter code and were colored according to biophysical properties in yellow (hydrophobic), blue (positively charged), or green (hydrophilic). (**D**) Translocation activities of the TP1170 nanobody constructs carrying the 11 different signal peptides. SecYEG and SecA were co-expressed from plasmids (two technical replicates). (**E**) Translocation assays of the TP1170 nanobody carrying the two best signal peptides, YncJ and OmpA(extCore), in combination with SecYEG WT or the super-active SecY variant I408G variant (technical triplicates). As control, the TP1170 nanobody carrying the standard OmpA signal peptide was used. Kinetic data are shown as mean, error bars indicate the SEM.

While our screen had identified several mutations that globally increase translocation activity of the SecY pore, translocation is additionally dictated by the sequence of individual substrates and their corresponding signal peptides, which determine translocation pathway and efficiency (*45*, *46*). We hypothesized that ProSecCO could be harnessed to optimize translocation of specific proteins. To test this hypothesis, we chose a biotechnologically relevant secreted protein, the anti-mouse-IgG nanobody TP1170 (*47*), as target. We selected 11 different signal peptides, including various natural sequences from *E. coli* (*45*) and *Corynebacterium glutamicum* (*48*), as well as synthetically derived sequences (*48*–*50*), which we N-terminally fused to the TP1170 nanobody carrying a C-terminal high-affinity complementation tag (Fig. 5, B to C; table S1). Notably, translocation activities ranged almost 70-fold between the weakest (PorB) and the strongest signal sequences, YncJ and OmpA(extCore) (Fig. 5D). More importantly, translocation of TP1170 with the strongest two signal peptides, OmpA(extCore) and YncJ, could be further improved up to threefold when swapping SecY WT with the super-active SecY variant I408G that we had identified earlier in our library screen (Fig. 5E). Altogether, these experiments demonstrated that ProSecCO is able to identify optimized signal peptide-substrate combinations that act synergistically with enhanced SecYEG pores, thereby enabling simultaneous engineering of both the Sec machinery and substrate proteins to improve translocation efficiency.

## DISCUSSION

SecYEG is the central hub for protein partitioning and strictly essential for living cells, which makes it a focal point of research, but also difficult to assess the function of SecYEG mutants in vivo. In this study, we developed ProSecCO a cell-free system to produce, study and engineer SecYEG and its associated components in vitro. This cell-free approach decouples SecYEG activity from in vivo function, which allows to characterize even potentially lethal (i.e., non-active or super-active) SecYEG variants. ProSecCO enables direct testing of hundreds of genetic constructs without the need for laborious molecular cloning, protein purification and in vitro reconstitutions, thereby allowing an unprecedented increase in throughput compared to state-of-the-art approaches [∼two orders of magnitude (*21*, *24*, *41*, *51*)]. At the same time, ProSecCO provides substantially improved high-resolution quantitative data over a much broader dynamic range compared to current methods. This holds especially true for translocation assays, which are highly robust, while insertion assays show a lower signal-to-noise ratio, requiring more stringent experimental control. Furthermore, the bottom-up reconstruction of the SecYEG pore in blank vesicles provides a platform to conveniently assess the effects of auxiliary Sec components, such as YidC, SecA and SRP/SR, which enables direct investigations on the interactions of these proteins, while minimizing the risk of pleiotropic effects from complex in vivo studies.

Using ProSecCo to screen a library of ∼300 SecY variants, we nearly quadrupled the number of SecY variants described over last three decades. We identified several novel variants with increased translocation activity and – for the first time – also a variant with enhanced insertion activity. Overall, we report 77 different SecY variants with at least twofold increased translocation activity that are distributed over almost 40 distinct sites of the protein. Although our mutagenesis was focused on selected sites in the protein, the surprisingly high frequency with which mutations yield a super-active translocation phenotype suggests that SecY did not evolve for maximum translocation activity, but rather stringent membrane integrity and proof-reading (*52*). In contrast, we observe much lower impact of SecY mutations on insertion activity, identifying only one variant of 30% improved activity. This raises the question whether insertion activity has been already evolutionarily optimized, or whether SecY has been simply under sampled in respect to insertion activity in the field.

Our study provides a proof-of-concept for the in vitro production and insertion of high-quality membrane proteins and for rapid prototyping of efficient protein translocation across membranes. These insights will be valuable for future efforts to boost membrane-protein secretion and production in both natural and synthetic cell platforms. In sum, ProSecCO overcomes longstanding obstacles in Sec-machinery research, establishes a robust framework for dissecting Sec function, and opens new avenues for biotechnological and synthetic-biology applications (*53*).

## MATERIALS AND METHODS

### Chemicals and reagents

The library was ordered from GenScript, primers were ordered from Eurofins and gBlocks from TWIST Biosciences. Lipids were ordered as chloroform solutions (Avanti). The Nano-Glo HiBiT Extracellular Detection System, The Nano-Glo HiBiT Blotting System and QuantiFluor dsDNA System were purchased from Promega. For plasmid cloning, Q5 High-Fidelity DNA Polymerase 2x Master Mix, NEBuilder HiFi DNA Assembly Master Mix, DpnI, KLD Enzyme Mix and NEB Turbo *E. coli* were supplied by New England Biolabs. The NucleoSpin Plasmid miniprep and PCR clean up kits were supplied by Macherey-Nagel. PURE CFPS systems were purchased from Hölzel Diagnostika Handels GmbH (PUREfrex 1.0, Gene Frontier) or from New England Biolabs (PURExpress). Murine RNase inhibitors were purchased from New England Biolabs. Amino acids and tRNAs for lysate reactions were supplied by biotechrabbit (RTS Amino Acid Sampler) and Roche Diagnostics (tRNA, from E.coli MRE 600), respectively. Enzymes and reagents which were indicated for storage in ultra-low temperature freezers (including PUREfrex and PURExpress) were stored at −70°C to reduce energy consumption.

### Construction of pSecYEG

To synthesize the three *E. coli* Sec core proteins, SecY, SecE and SecG, we constructed a genetic base construct, the pSecYEG plasmid. It was designed for high expression of the core *E. coli* translocon from a single operon (fig. S2A). To ensure compatibility with common *E. coli* CFPSs and avoid toxicity issues during cloning we used the T7 promoter. While the relative expression levels were not optimized for translocation activity, we tuned the translation initiation rate of the core components to match the SecYEG translocon stoichiometry (*54*). The operon was concluded with two consecutive terminators: L3S2P21 (*55*) and T7 terminator.

### Protein purifications of SUMO-dark and 11S

SUMO-dark and 11S proteins were purified via N/C-terminal His-tags. Plasmids under T7 control were transformed into chemically competent BL21 (DE3) cells. The whole plate of transformants was used to inoculate 1 L TB medium with the appropriate antibiotics and grown in 5 L baffled Erlenmeyer flasks at 25°C for 24 h. The cells were not induced as the leakiness of the cells ensured high levels of protein production. Cells were harvested by centrifugation and resuspended in buffer D (20 mM Tris-HCl pH7.5, 50 mM KCl) + 20 mM imidazole supplemented with 10 μg/mL DNase I (Thermo Fisher) and 0.1 mM PMSF. Cells were lysed using a homogenizer (EmulsiFlex-B15, Avestin) and proteins extracted from the cleared lysate using Protino Ni-NTA agarose in gravity flow columns (Protino, Machery-Nagel). Proteins were eluted in buffer D + 500 mM imidazole and the imidazole removed using PD-10 desalting columns (Sigma-Aldrich) eluting into buffer D. Proteins were concentrated using amicon spin concentrators (3 kDa MWCO, Sigma-Aldrich), snap-frozen in liquid nitrogen and stored at −70°C.

### Liposome preparation

Large unilamellar vesicles (LUVs) were prepared by lipid swelling and subsequent extrusion. 5 mg phospholipids in chloroform were added to a glass test tube and dried under a stream of nitrogen followed by applying vacuum for at least 1.5 h. The standard lipid composition was DOPC:DOPE:DOPG 40:30:30 molar ratio. The vacuum was released with nitrogen and 500 μL of inner aqueous solution (IA) added at room temperature. 500 μL of IA contained 51 μL 11S protein (LgBiT, Nano-Glo HiBiT Extracellular Detection System, Promega) and 12 μL 50% glycerol (w/v) in 17.5 mM HEPES pH7.2. Alternatively, to the commercial LgBiT stock, 7 μM purified 11S protein was used. In that case, 51 μL 50% glycerol (w/v) was added instead of the commercial LgBiT stock to adjust osmolarity. The suspension was incubated at room temperature for 30 min with occasional vortexing. After 5 freeze-thaw cycles between liquid nitrogen and a room temperature water bath, the suspension was extruded 11 times through a 0.1 μm polycarbonate membrane (GE Healthcare) using a mini-extruder (Avanti Research). Liposomes were washed 3 times by centrifugation for 1 h at 45,000 g and 4°C, removing the supernatant and resuspension in liposome wash buffer (6.3% glycerol, 17.5 mM HEPES pH7.2). Finally, reporter LUVs were resuspended in 50 μL liposome wash buffer yielding about 42 mg/mL lipids and stored at 4°C for up to 5 days.

Empty “competitor” LUVs without encapsulated proteins were prepared similarly but instead, 5 mM HEPES pH7.2 was used as IA and for resuspension while freeze-thawing was omitted. Empty LUVs were concentrated to 60 mg/mL by centrifugation, snap-frozen in liquid nitrogen and stored at −70°C.

### Nanoluc translocation and insertion assays

For real-time kinetic translocation assays, a nanoluc based in vitro assay was adapted for the use in our system (*14*). Briefly, reporter liposomes contain the catalytically inactive 11S (LgBiT) variant of nanoluc which is activated by a high affinity complementation tag (pep86) or alternatives of it (*15*). Upon successful translocation of a reporter protein with the C-terminal complementation tag into the liposome lumen, a luminescent signal is emitted and detected in a plate-reader. To eliminate unspecific luminescence due to liposome breakage, a protein tagged with the dark peptide, in our case SUMO-dark, is added to the bulk solution (*14*).

To ensure proper translocon activity, the assay was divided into two phases: first the expression of the Sec system, followed by reporter expression and luminescence recording. The primary CFPS containing 60–150 μM SUMO-dark, 0.2 nM pSecYEG, 0.1 nM pSecA, 3.3 mg/mL blank competitor vesicles (without 11S; not added in lysates) and glycerol to adjust osmolarity (only lysates; measured with OM807, Loeser) was assembled on ice. Per 10 μl CFPS, 1 μL 42 mg/mL reporter liposomes were added on top and incubated in PCR tubes for 3 h at 30°C. From this point, samples were not cooled beneath room temperature to avoid precipitation of hydrophobic proteins. In the meantime, reporter CFPS was prepared: 60–150 μM SUMO-dark, 0.4 nM reporter DNA, glycerol to adjust osfmolarity (only in lysates) and 1x furimazine (Promega). After 3 h of Sec expression, primary and reporter CFPS were mixed at room temperature at equal ratio, and 9 μl each were dispensed into a 384-well plate (#784904, Greiner) in technical triplicates. The plate was sealed with transparent cover foil, centrifuged briefly (400 g) and immediately measured in a plate-reader (Infinite 200 Pro, Tecan) at 30°C. Alternatively to co-expression with pSecYEG, SecA was pre-expressed in a separate CFPS reaction and added to the reporter CFPS at a ratio of 1:20 (1:40 final dilution) in order to prevent resource competition.

Insertion of integral membrane proteins was measured according to the same principle as translocation. In this case, SecA was omitted and a membrane protein reporter (i.e. PR(−1tm)-pep104 or LepB-pep86) was used. To account for CFPS resource competition between the reporter and the Sec constructs, non-tagged full-length proteorhodopsin (pTE5620) was expressed instead of pSecYEG as a spontaneous integration control plasmid that does not convey insertase activity. If applicable, membrane bound auxiliary components, like the YidC insertase, were co-expressed with pSecYEG at 0.1 nM plasmid concentration. Soluble components, like SRP and SR, were usually expressed separately and added to the reporter CFPS at a ratio of 1:20 (1:40 in the final insertion reaction).

Luminescence data was collected in technical triplicates. At each collected time point a luminescence slope (RLU/s) was calculated with a linear regression including the previous and subsequent time point (3-point calculation). In contrast to previous reports using a luminescence translocation assay and purified reporter proteins, we 1) did not perform a background subtraction and 2) used the maximum slope (max. slope) as the parameter for translocation/insertion activity because of two reasons: 1) When expressing the proOmpA-pep99 reporter alone and without SecYEG we only observed a negligible background signal of about 0.5%. 2) Due to the constant expression of reporter in CFPS, the maximum luminescence signal does not reflect 100% reporter import but rather the limitation of consumed furimazine. It should therefore not be used to calculate the time of half maximum signal (t_50%_) in our cell-free system.

### SecY library translocation and insertion assays

Translocation and insertion activities for the library of pSecYEG variants were examined in luminescence assays. The library was ordered as arrayed clonal and purified plasmids (Genscript), dissolved in water and stored in a 384-well Echo source plate (384 pp., Beckman Coulter). The concentration of each variant was measured with the QuantiFluor dsDNA System (Promega) by transferring 1 μL each into wells of 96-well plates using an Echo 525 liquid handler (Beckman Coulter), adding 200 μL QuantiFluor reagent and measuring fluorescence in a plate-reader (Infinite 200 pro, Tecan). Assay plates were prepared by transferring 0.6 fmol DNA of each variant into separate wells of a 384-well plate (#784904, Greiner) using an Echo 650 T liquid handler (Beckman Coulter). A sufficient amount of assay plates for all experiments and replicates was prepared at the same time, sealed with adhesive PCR plate foils and stored at −20°C.

For a luminescence assay, one plate containing all variants and control plasmids was thawed, the cover foil removed and the water evaporated by applying vacuum for 15 min using a desiccator. On ice, 3 μL primary CFPS containing all necessary components (except pSecYEG) including reporter liposomes was added to each well using a multi-channel dispenser (VOYAGER, Integra). For translocation experiments, the mix contained 0.1 nM pSecA. This resulted in a pSecYEG concentration of 0.2 nM in the primary CFPS. The plate was sealed with a transparent cover foil, mixed by orbital shaking, briefly centrifuged at 400 g and then incubated at 30°C for 3 h. After this, 3 μL reporter CFPS was added per well. The plate was sealed, mixed, centrifuged and luminescence kinetics immediately measured for 2 h at 30°C in a pre-heated plate-reader (Infinite 200 Pro, Tecan). Reporter CFPS contained 0.4 nM proOmpA-pep99 (translocation) or 0.4 nM PR(−1tm)-pep104 reporter DNA (insertion).

Library assay replicate data for translocation and insertion was all collected on separate days and processed. At each collected time point a luminescence slope was calculated with a linear regression including the previous and subsequent time point (3-point calculation). The maximum raw luminescence (trans/insert_highest_value) and slope value (trans/insert_peak_slope) are reported in (data S1). In order to correct for assay variance between days, the average global peak_slope for every library member was divided by the average of the global peak_slope of all replicates (resulting in a minor adjustment since the daily variance was low). Rarely, there was the appearance of an outlier replicate which was removed from the normalized data, defined by a > 25% difference from the average of all replicates, resulting in removal of <10% of normalized values. The normalized average was recalculated using the filtered values. This filtering step was not performed for the insertion data because there were 2 replicates. Finally Log2 fold-change was calculated compared to R22R_CGC for the WT reference. Please refer to (data S1) for all raw and normalized values.

### In vitro assembly of linear SecY-mNeonGreen fusion constructs

A subset of SecY library mutants was genetically fused to C-terminal mNeonGreen to assess their expression and folding. To quicken the production of such constructs, we adapted an entirely in vitro workflow to assemble DNA constructs and produce linear expression templates (LETs) via PCR (*56*). Briefly, DNA fragments with BsaI sites were produced by PCR, annealed via Golden Gate assembly to form intact plasmids, and these were directly used as PCR templates to then produce LETs harboring the whole operon including: T7 promoter, *secY*-mNeonGreen, *secE*, *secG* and the two terminators.

A parental plasmid based on pSecYEG with a C-terminal fusion of mNeonGreen to SecY (pTE5644) was constructed by traditional Gibson cloning. From this, a linear backbone with flanking BsaI sites was produced by PCR (Q5 High-Fidelity, NEB) using primers which excluded the region starting with the T7 promoter and ending with the C terminus of secY (primers oMM-171 atactacGGTCTCCctgaaaggctacggcc, oMM-169 atactacGGTCTCCccttgcatgcaccaacc). The fragments with BsaI sites to insert different SecY mutants were producted by PCR (primers oMM-165 atactacGGTCTCCaaggagatggcgccc, oMM-167 atactacGGTCTCCtcaggttcgccttcttca) amplifying the region from T7 promoter to the end of secY using different library variants as templates. To avoid carry-over and amplification of parental plasmids, only 0.005–0.1 ng template plasmid were used per 25 μL PCR reaction, and residual template DNA was removed by incubating the PCR products with DpnI at 37°C over night. Inserts and backbone were purified using a PCR clean-up kit (Monarch, NEB) and then assembled in 5 μL Golden Gate reactions (NEB) containing: 1x T4 DNA Ligase buffer, 2 U/μL BsaI-HFv2, 20 U/μL T4 DNA Ligase, 2.5 nM backbone and 5 nM insert. Golden Gate assemblies were incubated at 37°C for 45 min, heat inactivated for 5 min at 60°C and directly used as PCR template (Q5 High-Fidelity, NEB) at a final dilution of 1:1000 (oMM-173 gcgtagaggatcgagatct, oMM-174 gtcccattcgccaatcc). The resulting LETs were purified via PCR clean-up and verified by agarose gel electrophoresis and Sanger sequencing. LETs were used in PURE reactions in the same molar concentrations as plasmid constructs.

### Fluorescence assays to measure membrane protein expression and folding

Expression and membrane insertion of the membrane proteins SecY and PR was assessed using C-terminal mNeonGreen fusions as folding indicators. This method assumes that only fully translated and non-aggregated proteins allow for mNeonGreen folding resulting in fluorescence (*32*, *33*). 5 μL PURE reactions containing 0.4–0.5 nM DNA (plasmid or LET) and 3.3 mg/mL empty liposomes were assembled were assembled on ice and transferred into a 384-well plate (#788096, Greiner). Fluorescence (excitation at 485 nm, emission at 515 nm) was measured at 30°C in a plate-reader (Infinite 200 Pro, Tecan) with 20 s orbital shaking (3 mm amplitude) every 3 minutes.

### Protease protection assays

The successfully translocated and inserted reporter proteins were shielded form proteolytic digest and confirmed in protease protection assays (*9*). CFPS (15 μL) with 0.5 nM pSecYEG and 0.25 nM secA was assembled on ice and mixed with 1.5 μL 42 mg/mL freshly prepared and osmolarity matched empty liposomes (without 11S). The mix was incubated at 30°C for 3 h and then, at room temperature, topped up with 15 μL fresh CFPS containing 0.2 nM reporter DNA (with pep86 tag). The reaction was incubated at 30°C for 2.5 h to ensure successful translocation, transferred on ice and then gently mixed with 1x CutSmart (NEB) osmotically matched with glycerol (measured with OM807, Loeser). The mix was incubated on ice with 100 μg/mL Proteinase K (PK) and then precipitated with 7.5% ice cold TCA (w/v). As controls, samples were either not treated with PK or with PK and 1% Triton X-100. The precipitated samples were incubated on ice for another 30 min and then pelleted for 10 min at 17,000 g and 4°C. After removing the supernatant, the pellet was washed with 500 μL ice cold acetone and centrifuged again and the supernatant was removed. The pellets were dried for 5 min at 37°C, dissolved in 20 μL 2x Laemmli buffer and stored at −20°C. After SDS PAGE, the proteins were transferred on a nitrocellulose membrane and visualized using the Nano-Glo HiBiT Blotting System (Promega) and (ChemiDoc MP, BioRad).

### Dynamic light scattering (DLS) analysis of liposomes

The distribution of hydrodynamic diameters and polydispersity index (PDI) of liposomes after translocation and insertion reactions were analyzed using a Zetasizer Nano S90 (Malvern Panalytica GmbH). 10 μL sample were mixed with 190 μL PBS and measured in three technical replicates. Z-average values were calculated as averages and derivations of the main peaks.

### Cryogenic transmission electron microscopy (cryo-TEM) of liposomes

Cryo-TEM micrographs of liposomes were acquired using a FEI Titan Krios G4 TEM (Thermo Scientific). 3 μL sample were applied to a Quantifoil or lacey carbon coated TEM grid which had been glow discharged in a Diner Nano oxygen plasma cleaner (Diener electronic) briefly before. Excess sample solution was removed with a filter paper and the grid plunged into liquid ethane using a Vitrobot Mark V (Thermo Fisher). Imaging was done under cryogenic conditions with an acceleration voltage of 300 kV. Micrographs were acquired under low dose conditions using a 4 k Direct Electron Detection Camera (Gatan K3).

### Lysate preparation

Cell extracts for lysate based cell free expression were prepared from BL21 Gold (DE3) cells either following a protocol for disruption by homogenization (*57*) or autolysis (*58*). In short, cells were grown to mid-log phase, harvested by centrifugation and resuspended in S30A buffer followed by lysis using a high-pressure homogenizer (EmulsiFlex-B15, Avestin) or by freeze-thawing and vortexing for cells bearing the pAD-LyseR plasmid. Cell debris was removed by centrifugation for 1 h at 12/30/50/80 or 105 k g. Autolysates were subjected to a run-off at 37°C for 2 h before snap-freezing and storing lysates at −70°C.

### Cell free lysate reactions

Apart from using PURE kits, cell free reactions were performed in *E. coli* lysate based CFPS systems. The energy mix containing all necessary factors to ensure high expression was prepared as previously described (*59*) with some optimizations (*60*). Cell free reactions were assembled on ice with the final composition being: 33% autolysate or 27% homogenizer extract, 0.5 mM aa’S (except 0.42 mM Leu), 53 mM HEPES, 1.04 mM ATP and GTP, 0.63 mM CTP and UTP, 0.040 mg/mL tRNAs, 0.13 mM CoA, 0.17 mM NAD, 0.57 mM cAMP, 0.068 mM folinic acid, 1.00 mM spermidine, 25 mM 3-PGA, 0.67 U/μL murine RNase inhibitors (NEB). Mg-glutamate, K-glutamate, DTT and PEG were individually titrated for each lysate. Usual concentrations were: 6 mM Mg-glutamate, 80 mM K-glutamate, 1.5 mM DTT and 3% PEG-8000. DNA was added between 0.1 and 1 nM but usually 0.2 nM. For constructs based on expression from the T7 promoter, 0.25 μL T7 polymerase (NEB) was added per 10.5 μL reaction. Reactions were incubated in PCR tubes at 30°C. For Fluorescence and luminescence measurements, the reactions were transferred into 384-well plates (#784076 or #784904, Greiner) and measured in a plate reader (Infinite 200 Pro, Tecan).

### Measurement of transcriptional and translational activity of *E. coli* lysates

Transcription and translational outputs of *E. coli* cell free lysates were measured expressing a fusion construct encoding sfGFP and Pepper aptamer (*61*). Standard CFPS reactions with 1 nM pTE5422 (Addgene ID 229031) were assembled on ice and supplemented with 10 μM HBC620 (MedChemExpress, Monmouth Junction, NJ, USA, catalog no.: HY-133520). 10 μL reactions were transferred into 384-well plates (#784076, Greiner), sealed with a transparent cover foil, centrifuged briefly at 400 g and incubated in a pre-heated plate-reader (Infinite 200 Pro, Tecan) at 30°C. Pepper and sfGFP expression was followed for 3 h measuring fluorescence at 575/620 nm and 485/528 nm respectively. To compare the lysates, end-point values were used for sfGFP and the maximum values for the Pepper aptamer.

### nFCM measurement of native *E. coli* vesicles

Native vesicle contents of *E. coli* cell free extracts were measured by nano-flow cytometry (nFCM) using a NanoAnalyzer (NanoFCM Co., Ltd., Nottingham, UK). The device was calibrated with 2.10 x 10^8^ particles/mL or 2.16 x 10^8^ particles/mL 250 nm QC beads (NanoFCM Co.) and monodisperse silica beads of four different diameters (68, 91, 113 and 155 nm, NanoFCM Co.) as size reference standards. Samples were diluted in freshly filtered TE buffer which was also measured separately for background subtraction. The measurements were performed for 1 min at a sampling pressure of 1.0 kPa. The NanoFCM software (NF Profession V2.3) was used to calculate particle concentration, mean size and size distribution.

## Supplementary Materials

### The PDF file includes:

Figs. S1 to S9 Table S1 Legends for data S1 to S5 References
