# Supplementary material for: Functional mapping and engineering of the Sec translocon unlocked by a cell-free system
Source: Sci Adv. 2026 Sep 2;12(36):eaej0987. doi: 10.1126/sciadv.aej0987 (PMC13537251; doi:10.1126/sciadv.aej0987)
Supplement: Supplementary file 1 [file sciadv.aej0987_sm.pdf]

Science Advances
AAAS

Supplementary Materials for

Functional mapping and engineering of the Sec translocon unlocked by a cell-free system

Markus Meier *et al.*

Corresponding author: Scott A. Scholz, scott.scholz@mpi-marburg.mpg.de;
Tobias J. Erb, toerb@mpi-marburg.mpg.de

Sci. Adv. **12**, eaej0987 (2026)
DOI: 10.1126/sciadv.aej0987

The PDF file includes:

Figs. S1 to S9
Table S1
Legends for data S1 to S5
References

Other Supplementary Material for this manuscript includes the following:

Data S1 to S5

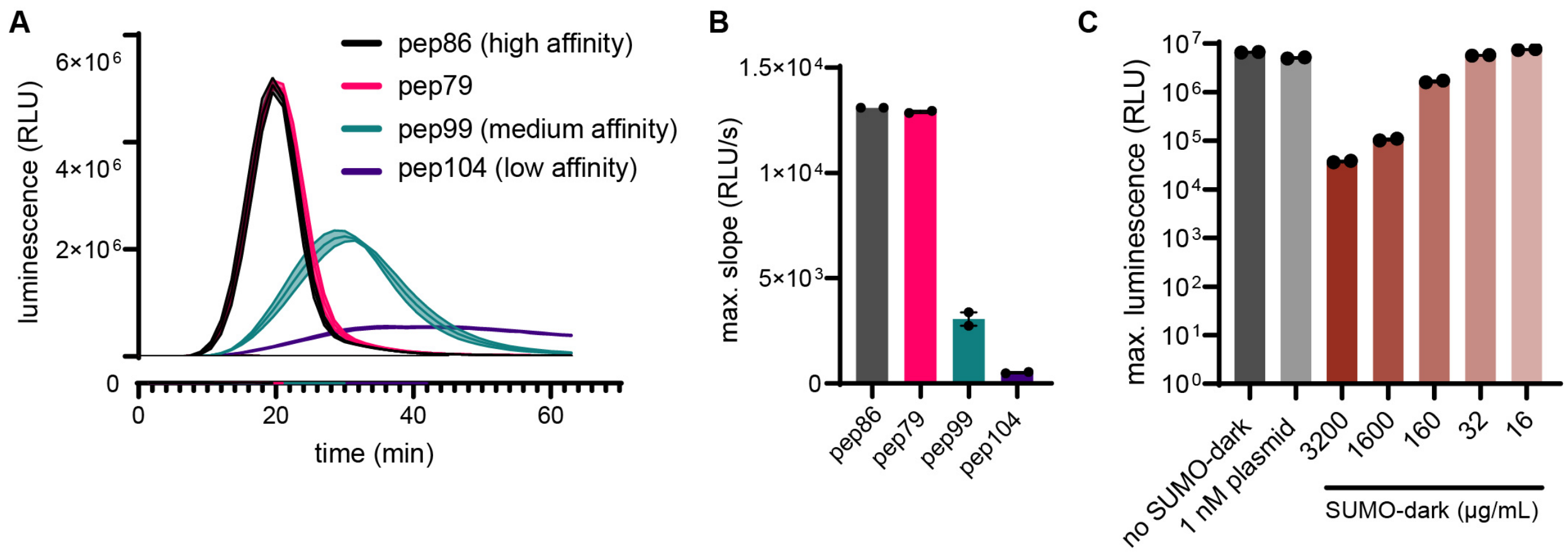

Fig. S1.

Adaption of a split nanoluc system for the use in CFPS. A) Bulk expression kinetic data of proOmpA reporters in S50 autolysate (11S and furimazine present in bulk solution; no vesicles). proOmpA constructs with high/medium/low-affinity complementation tags (pep86/pep99/pep104) with differing binding affinities to 11S were expressed from plasmids. B) Resulting maximum slope data. C) Titration of purified SUMO-dark protein in CFPS with proOmpA-pep86 expressed in bulk. Kinetic data are shown as mean of two technical replicates.

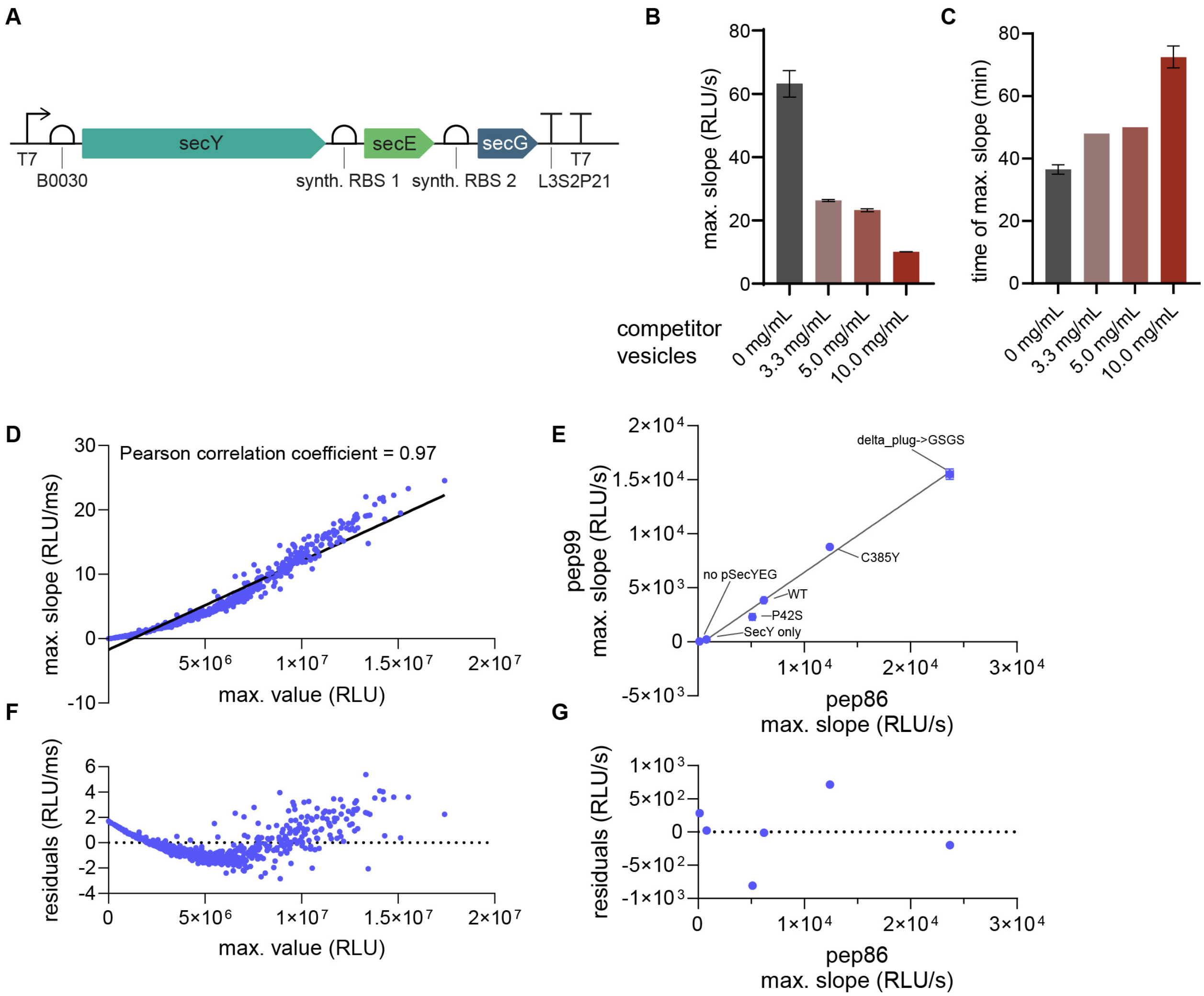

Fig. S2.

Development of the split nanoluc translocation assay for cell free expressed SecYEG. A) Genetic map of the *secYEG* operon of the pSecYEG construct for cell free expression of functional SecYEG. B) Titration of competitor vesicles (blank membrane; no 11S) in translocation assays using PUREfrex 1.0 and C) their influence on the assay life time. D) Linear regression of maximum luminescence against maximum slope values derived from translocation assay data. F) The respective residuals. Data points were taken from all three translocation replicates of the pSecYEG library. E) Translocation assay data comparing the luminescence output for proOmpA fused to the high (pep86) and medium affinity tag (pep99). Different SecY mutants of pSecYEG were used to compare the two tags. G) Residuals of the linear regression of E). Kinetic data are shown as mean of technical triplicates, error bars indicate the SEM.

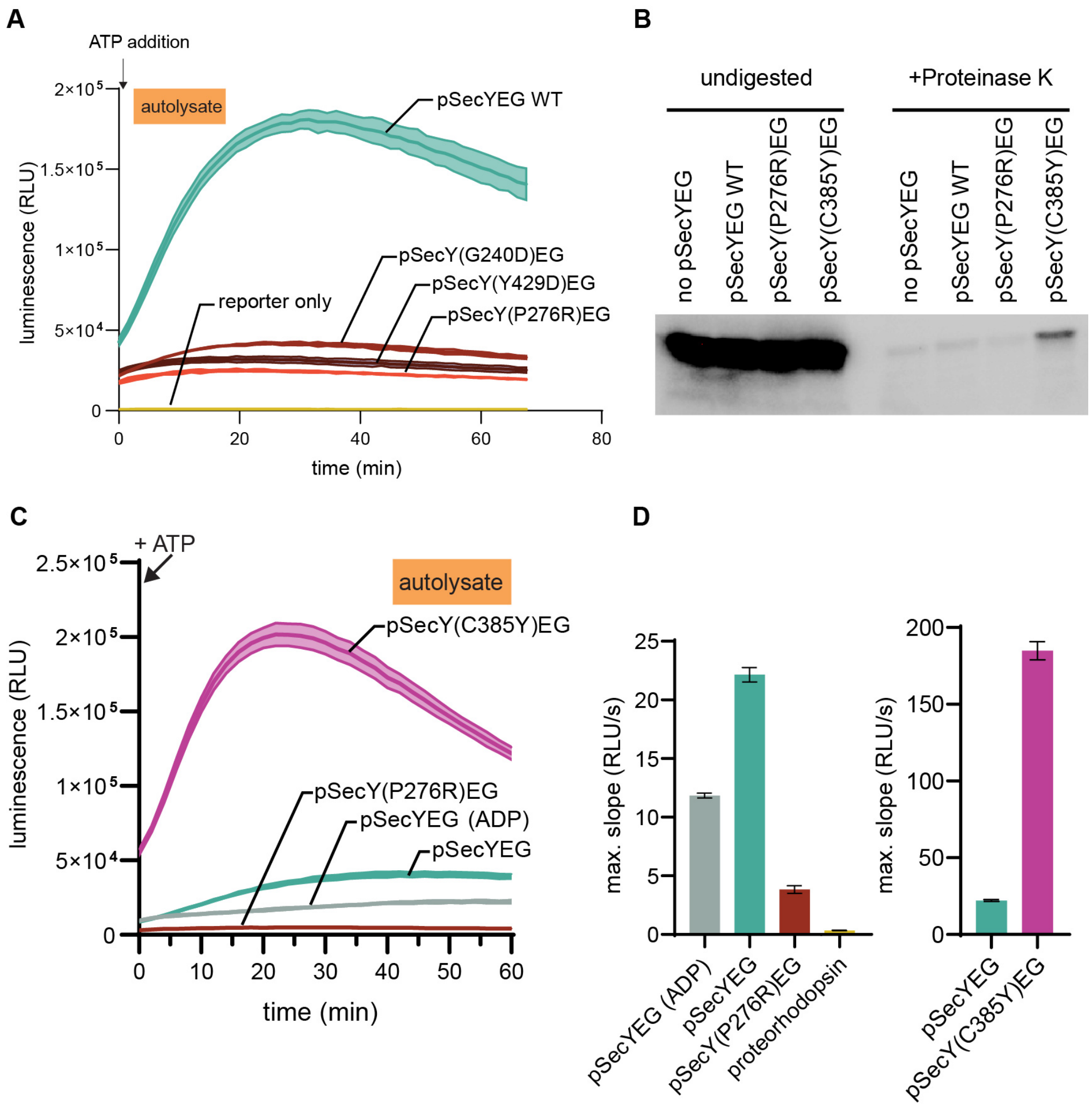

Fig. S3.

Validation of the translocation assay in *E. coli* S50 autolysate. A) Co-expression of pSecYEG together with the motor ATPase SecA and proOmpA-pep99 in S50 autolysate conveys an ATP dependent translocation signal. 20 mM ATP and 1x furimazine were added after 2.5 h of expressing all three plasmids at the same time. Common translocation deficient SecY mutants (G240D (*20*), Y429D (*20, 21*) and P276R (*19*)) do not respond to ATP addition. B) Protease protection assay for proOmpA-pep86 translocation through different mutants of the pSecYEG plasmid in lysate. The protein band corresponds to non-digested/protected proOmpA-pep86 reporter. C) Translocation assay similar to a but testing a super-active SecY variants (C385Y (*12, 24*)) and the ATP dependence of SecA driven translocation. D) Maximum slope data of C). Kinetic data are shown as mean of technical triplicates, error bars indicate the SEM.

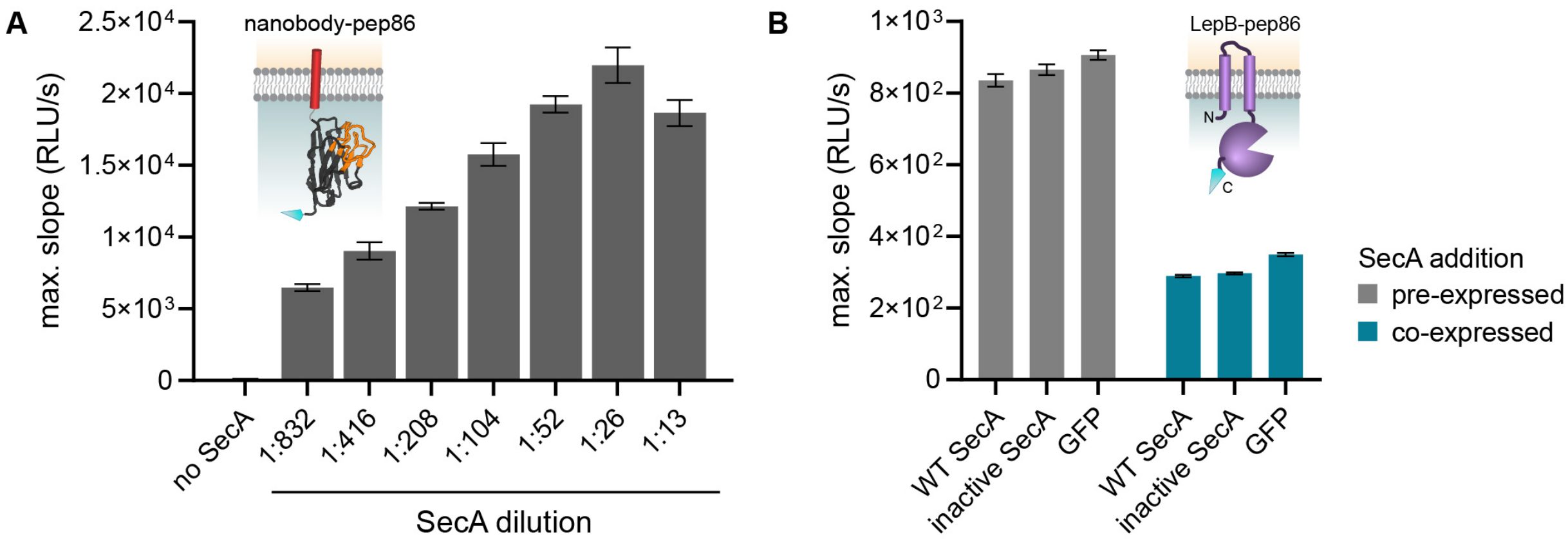

Fig. S4.

Titrating pre-expressed SecA for translocation experiments. A) Translocation assay in PUREfrex 1.0 adding different amounts of pre-expressed SecA to the reporter CFPS. Final dilutions in the resulting reaction mix are indicated on the x-axis. SecA was pre-expressed in PURE from 0.5 nM pSecA plasmid. SecYEG in the primary CFPS was expressed from 0.5 nM pSecYEG, and the reporter CFPS contained 0.4 nM pTE5636 reporter DNA (nanobody-pep86 with OmpA(extCore) signal peptide). Standard translocation reactions contained 1:40 diluted pre-expressed SecA to ensure saturation. B) Insertion/translocation assay to test the influence of SecA on LepB-pep86 insertion and translocation. To elucidate the effect of CFPS resource competition, SecA was either co-expressed with pSecYEG or pre-expressed separately and added to the reporter CFPS. GFP and SecA E210Q ('inactive') were used as negative controls (*62, 63*). Kinetic data are shown as mean of technical triplicates, error bars indicate the SEM.

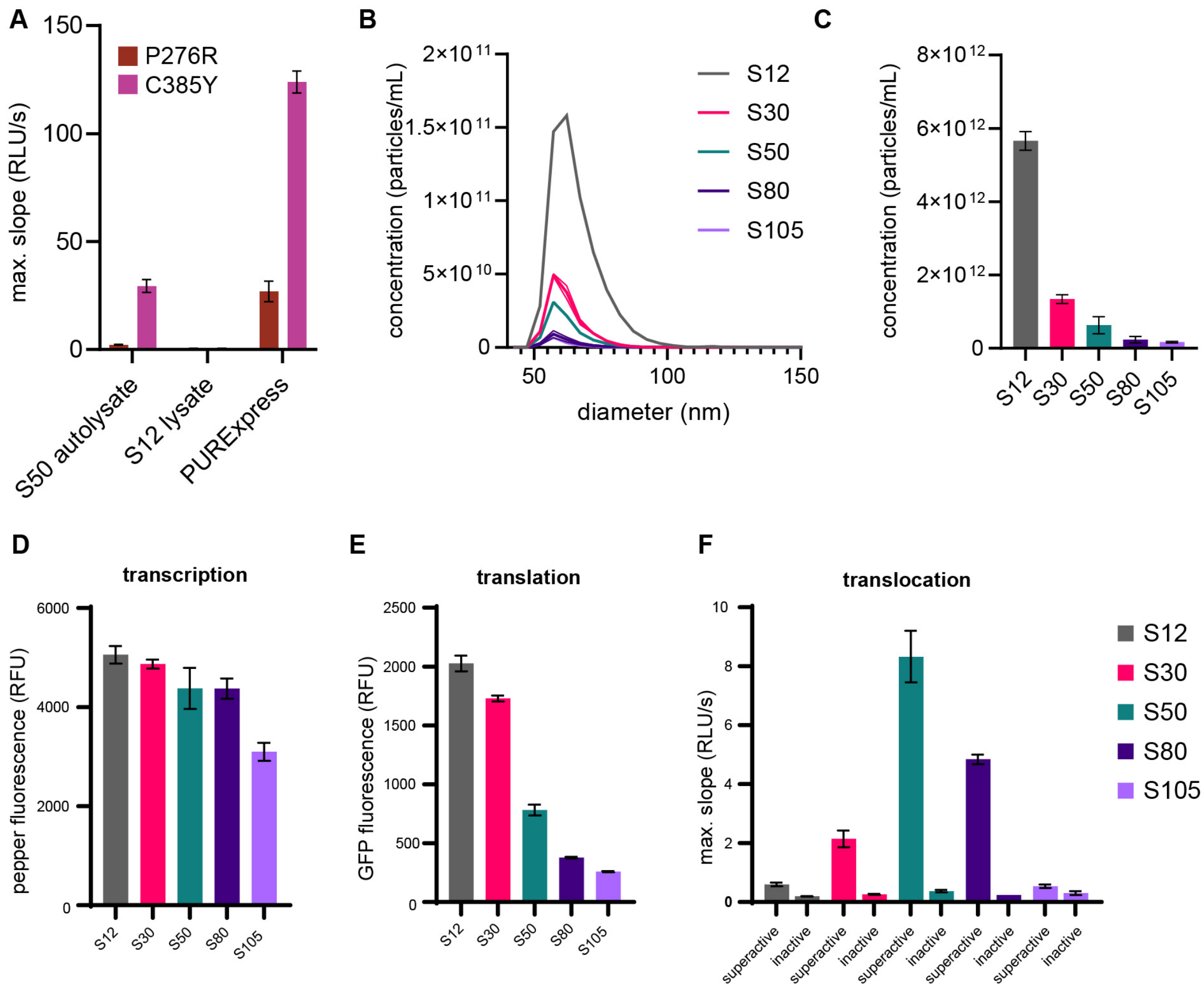

Fig. S5.

SecYEG dependent protein translocation in PURE and cell-free lysates. A) Translocation assays in three different *E. coli* based CFPS systems using two different variants of pSecYEG with an impaired (P276R (*19*)) and a super-active SecY mutant (C385Y (*12, 24*)). B) Particle size distribution of different five cell free lysates produced by homogenization and cleared at varying centrifugal speeds. C) Total concentration of vesicles in the lysates measured with nFCM. D) Transcription and E) translational activity were assessed using a sfGFP-pepper fusion construct and measuring fluorescence. F) Translocation assay using the C385Y (superactive) and P276R (inactive) SecY mutants. Kinetic data are shown as mean of technical triplicates, error bars indicate the SEM.

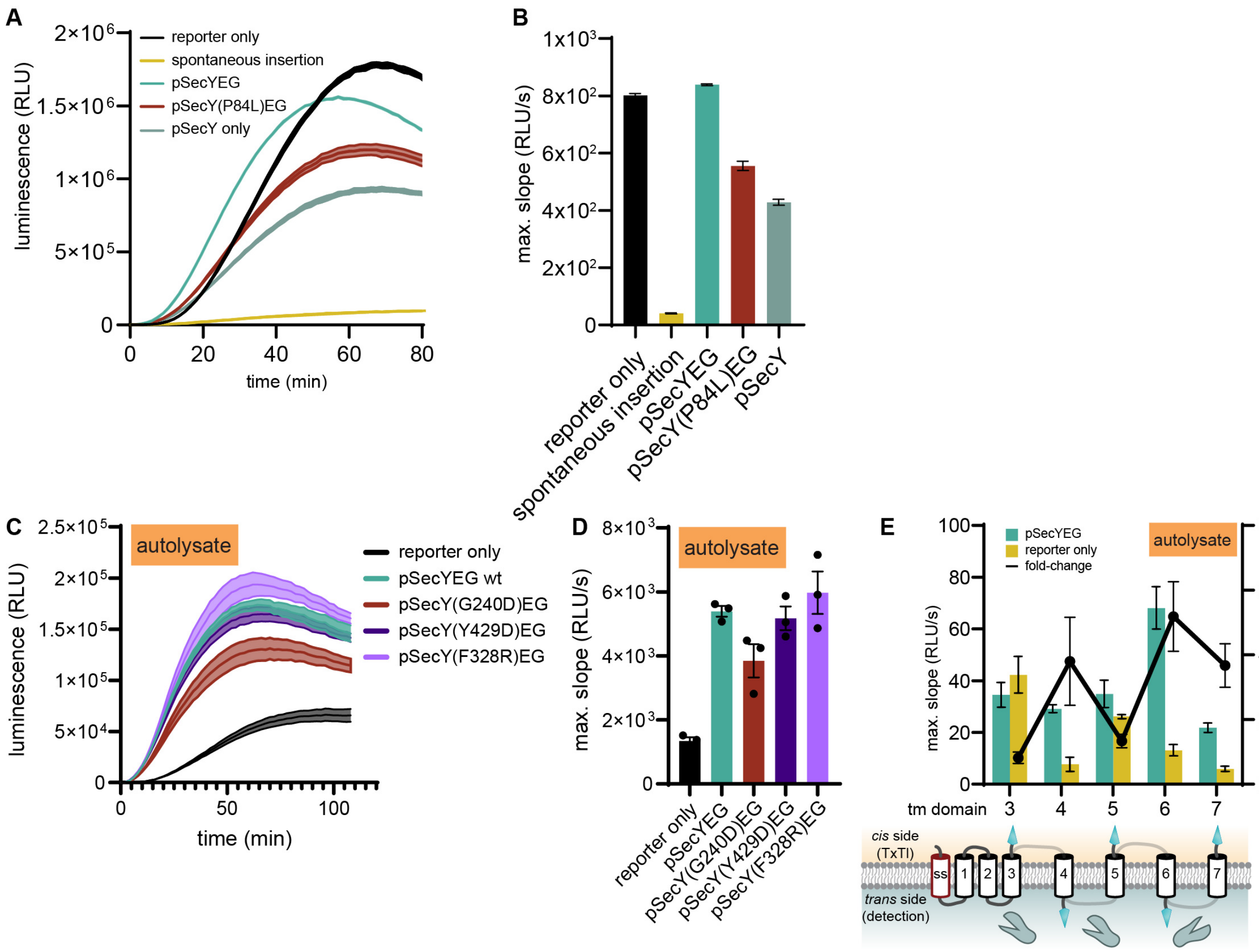

Fig. S6.

Membrane protein insertion assays. A) Insertion assay in PUREfrex 1.0 using the PR(-1tm)-pep104 reporter. In the "reporter only" control the reporter was expressed alone and without another plasmid compensating for resource competition with pSecYEG. In the spontaneous insertion control, a plasmid encoding native and non-tagged proteorhodopsin was expressed instead of pSecYEG to account for CFPS resource competition. B) Maximum slope values of A). C) Insertion assay in S50 autolysate using the same reporter. The G240D mutant has been reported to be deficient in insertion (*20*) while Y429D has been reported to have to effect on insertion (*20*). D) The respective maximum slope values for different SecY variants of pSecYEG. E) Assessment of directionality of insertion via SecYEG in S50 autolysate. Kinetic data are shown as mean of technical triplicates, error bars indicate the SEM.

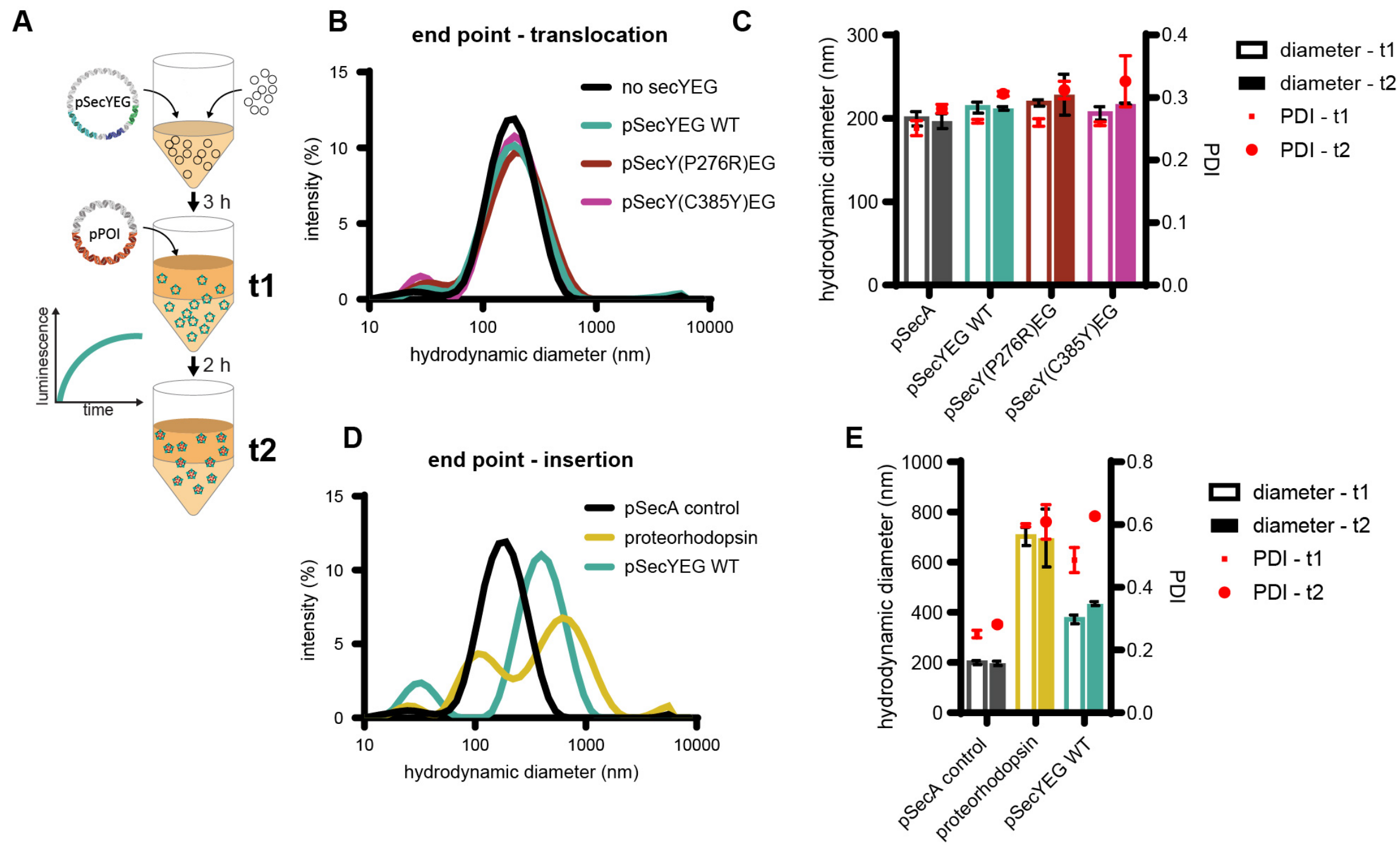

Fig. S7.

Reporter vesicle analysis during translocation and insertion experiments. A) During standard luminescence translocation (proOmpA-pep99) and insertion (PR(-1tm)-pep104) assays, vesicle samples were analyzed after the first expression phase establishing the SecYEG machinery (t1) and after the reporters had been synthesized (t2). B) DLS analysis at t2 after translocation experiments. C) Mean hydrodynamic diameters and polydispersity indices (PDI) at both time points for translocation. D) DLS analysis at t2 after insertion experiments. E) Mean hydrodynamic diameters and PDI at both time points for insertion.

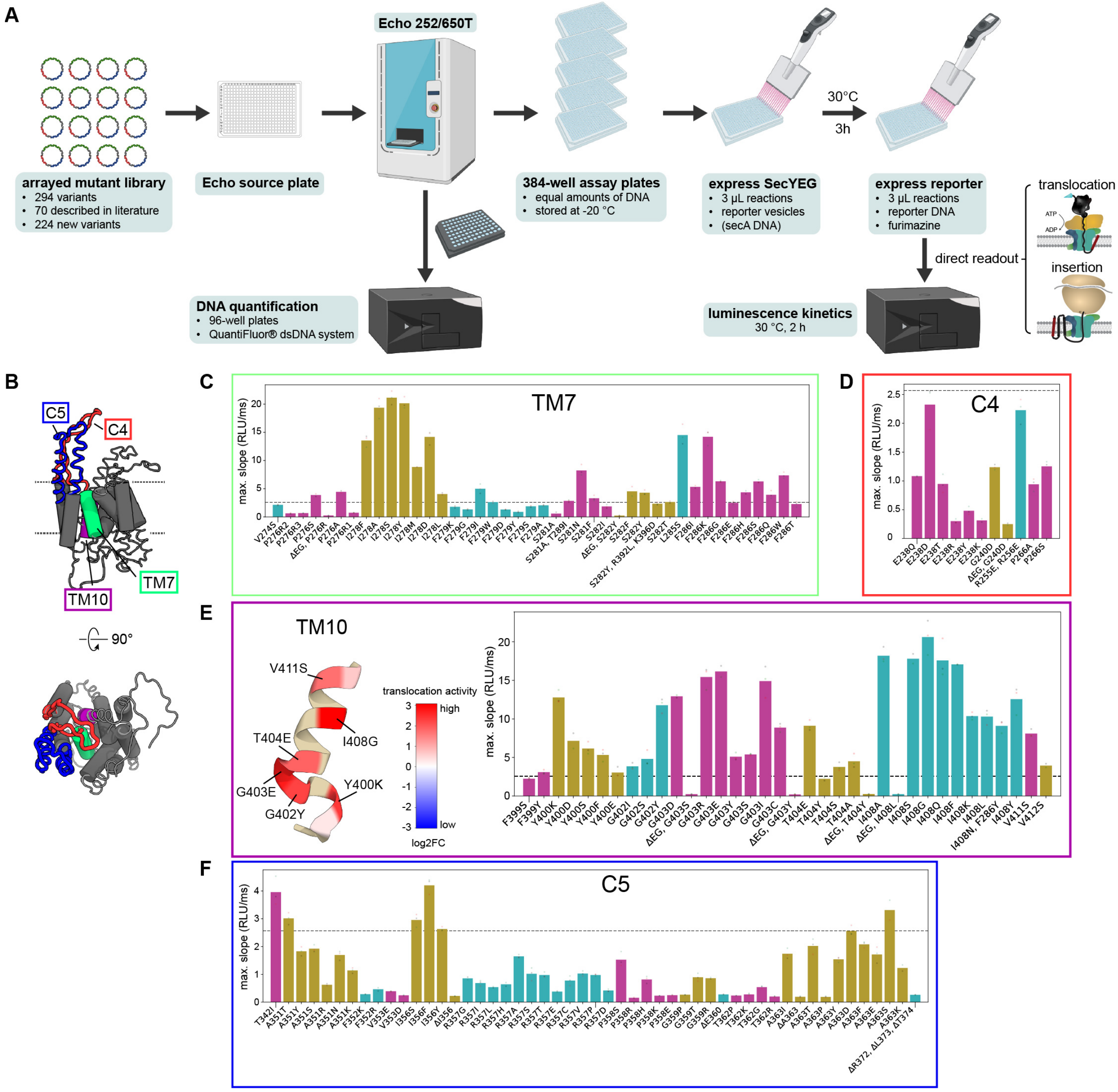

Fig. S8.

Translocation data of the pSecYEG library. A) Workflow of testing 294 variants of pSecYEG for their translocation and insertion activities (Created in BioRender. Meier, M. (2026) https://BioRender.com/x3y11gh). B) Schematic representation of the SecY structure. Highlighted are the cytoplasmic loops C5 (red) and C5 (blue), and the transmembrane domains TM7 (green) and TM10 (purple) (PDB: 5ABB). The individual translocation signals for each tested mutation within the different domains are shown in C) TM7, D) C4, E) TM10 and F) C5. The dashed horizontal lines indicate the WT activity. For TM10 the mutants with the highest translocation activity for each residue are color coded and plotted on the protein structure.

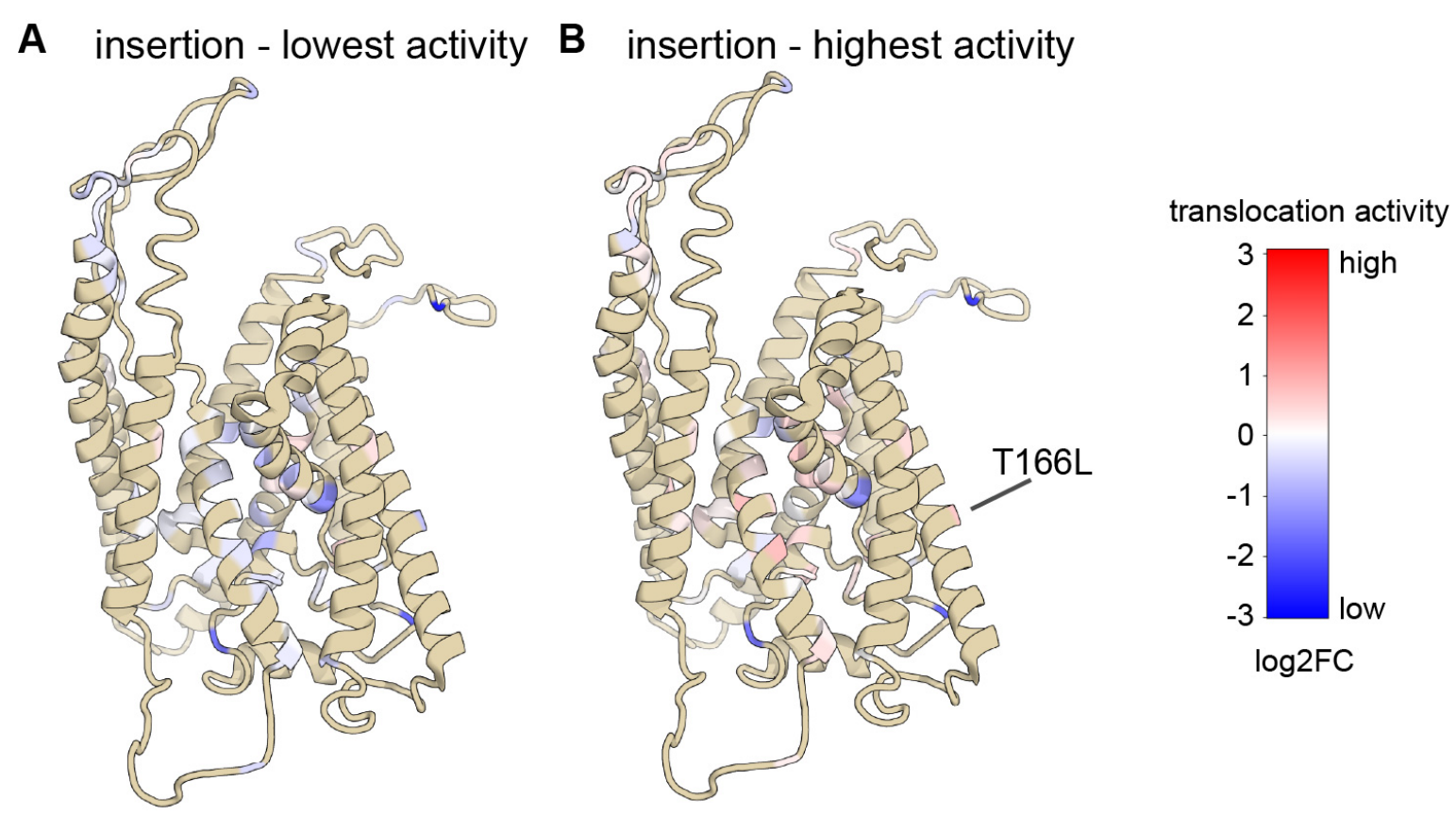

Fig. S9.

Insertion data of the pSecYEG library. A) Point mutants with the lowest and B) highest insertion activity obtained for each probed residue are plotted on the SecY structure according to their log2-fold-change over the WT activity measured in the library (PDB: 5ABB).

Table S1.

List of signal peptides used to translocate a nanobody through SecYEG.

name	organism	UniProt	reference	sequence
DsbA	*E. coli*	P0AEG4		MKKIWLALAGLVLAFSASA
PhoA	*E. coli*	P00634		MKQSTIALALLPLLFTPVTKA
PpiA	*E. coli*	P0AFL3		MFKSTLAAMAAVFALSALSPAAMA
TrbC	*E. coli*	P18473	Kavousipour, 2021 (*64*)	MKLSMKSLAALLMMLNGAVMA
YncJ	*E. coli*	P64459		MFTKALSVVLLTCALFSGQLMA
OmpA	*E. coli*	P0A910		MKKTAIAIAVALAGFATVAQAA
OmpA(A9N)	*E. coli*, deprecated mutant		Gennity, 1990 (*50*)	MKKTAIAINVALAGFATVAQAA
OmpA(extCore)	synthetic		Gennity, 1990(*50*)	MKKTAIVAIAIAIATIAVALAGFATVAQAA
PorB	*C. glutamicum*	H7C685	Jeon, 2024 (*48*)	MKLSHRIAAMAATAGITVAAFAAPASA
18F11_Jeon24	synthetic		Jeon, 2024 (*48*)	MAGYIRSAFALALAFTAFVFNPPLASA
ClustAlign	synthetic		Kavousipour, 2021 (*64*)	MKKSKALLALALALAASSFALASAFAAFA

Data S1. (separate file)
Extensive translocation and insertion results of the pSecYEG library.

Data S2. (separate file)
List of the plasmids used in this study.

Data S3. (separate file)
Explanation of the literature agreement test for library translocation results.

Data S4. (separate file)
Sequences (.gb files) of all plasmids generated in this work.

Data S5. (separate file)
Sequences (zipped .gb files) of all pSecYEG library plasmids generated in this work.

REFERENCES

1. Z. Cournia, T. W. Allen, I. Andricioaei, B. Antonny, D. Baum, G. Brannigan, N.-V. Buchete, J. T. Deckman, L. Delemotte, C. del Val, R. Friedman, P. Gkeka, H.-C. Hege, J. Hénin, M. A. Kasimova, A. Kolocouris, M. L. Klein, S. Khalid, M. J. Lemieux, N. Lindow, M. Roy, J. Selent, M. Tarek, F. Tofoleanu, S. Vanni, S. Urban, D. J. Wales, J. C. Smith, A.-N. Bondar, Membrane Protein Structure, Function, and Dynamics: a Perspective from Experiments and Theory. *J. Membr. Biol.* **248**, 611–640 (2015).

2. J. Yuan, J. C. Zweers, J. M. van Dijl, R. E. Dalbey, Protein transport across and into cell membranes in bacteria and archaea. *Cell. Mol. Life Sci.* **67**, 179–199 (2010).

3. A. Tsirigotaki, J. De Geyter, N. Šoštaric, A. Economou, S. Karamanou, Protein export through the bacterial Sec pathway. *Nat. Rev. Microbiol.* **15**, 21–36 (2017).

4. A. J. Harris, A. D. Goldman, The very early evolution of protein translocation across membranes. *PLoS Comput. Biol.* **17**, e1008623 (2021).

5. D. Akopian, K. Shen, X. Zhang, S. O. Shan, Signal recognition particle: an essential protein-targeting machine. *Annu. Rev. Biochem.* **82**, 693–721 (2013).

6. I. Kusters, A. J. Driessen, SecA, a remarkable nanomachine. *Cell. Mol. Life Sci.* **68**, 2053–2066 (2011).

7. E. Sobakinskaya, H. Krobath, T. Renger, F. Muh, Structural determinants of a permeation barrier of the SecYEG translocon in the active state. *Phys. Chem. Chem. Phys.* **23**, 25830–25840 (2021).

8. D. G. Knyazev, L. Winter, B. W. Bauer, C. Siligan, P. Pohl, Ion conductivity of the bacterial translocation channel SecYEG engaged in translocation. *J. Biol. Chem.* **289**, 24611–24616 (2014).

9. J. de Keyzer, M. van der Laan, A. J. Driessen, Membrane protein insertion and secretion in bacteria. *Methods Mol. Biol.* **390**, 17–31 (2007).

10. A. D. Silverman, A. S. Karim, M. C. Jewett, Cell-free gene expression: an expanded repertoire of applications. *Nat. Rev. Genet.* **21**, 151–170 (2020).

11. F. Katzen, T. C. Peterson, W. Kudlicki, Membrane protein expression: no cells required. *Trends Biotechnol.* **27**, 455–460 (2009).

12. H. Matsubayashi, Y. Kuruma, T. Ueda, In vitro synthesis of the E. coli Sec translocon from DNA. *Angew. Chem. Int. Ed. Engl.* **126**, 7665–7668 (2014).

13. N. Ohta, Y. Kato, H. Watanabe, H. Mori, T. Matsuura, In vitro membrane protein synthesis inside Sec translocon-reconstituted cell-sized liposomes. *Sci. Rep.* **6**, 36466 (2016).

14. G. C. Pereira, W. J. Allen, D. W. Watkins, L. Buddrus, D. Noone, X. Liu, A. P. Richardson, A. Chacinska, I. Collinson, A High-Resolution Luminescent Assay for Rapid and Continuous Monitoring of Protein Translocation across Biological Membranes. *J. Mol. Biol.* **431**, 1689–1699 (2019).

15. A. S. Dixon, M. K. Schwinn, M. P. Hall, K. Zimmerman, P. Otto, T. H. Lubben, B. L. Butler, B. F. Binkowski, T. Machleidt, T. A. Kirkland, M. G. Wood, C. T. Eggers, L. P. Encell, K. V. Wood, NanoLuc Complementation Reporter Optimized for Accurate Measurement of Protein Interactions in Cells. *ACS Chem. Biol.* **11**, 400–408 (2016).

16. R. Gupta, D. Toptygin, C. M. Kaiser, The SecA motor generates mechanical force during protein translocation. *Nat. Commun.* **11**, 3802 (2020).

17. I. Prabudiansyah, I. Kusters, A. Caforio, A. J. Driessen, Characterization of the annular lipid shell of the Sec translocon. *Biochim. Biophys. Acta* **1848**, 2050–2056 (2015).

18. C. van der Does, J. Swaving, W. van Klompenburg, A. J. Driessen, Non-bilayer lipids stimulate the activity of the reconstituted bacterial protein translocase. *J. Biol. Chem.* **275**, 2472–2478 (2000).

19. T. Tsukazaki, H. Mori, S. Fukai, R. Ishitani, T. Mori, N. Dohmae, A. Perederina, Y. Sugita, D. G. Vassylyev, K. Ito, O. Nureki, Conformational transition of Sec machinery inferred from bacterial SecYE structures. *Nature* **455**, 988–991 (2008).

20. H. G. Koch, M. Müller, Dissecting the translocase and integrase functions of the Escherichia coli SecYEG translocon. *J. Cell Biol.* **150**, 689–694 (2000).

21. T. Taura, Y. Akiyama, K. Ito, Genetic analysis of SecY: additional export-defective mutations and factors affecting their phenotypes. *Mol. Gen. Genet.* **243**, 261–269 (1994).

22. Y. Shimizu, A. Inoue, Y. Tomari, T. Suzuki, T. Yokogawa, K. Nishikawa, T. Ueda, Cell-free translation reconstituted with purified components. *Nat. Biotechnol.* **19**, 751–755 (2001).

23. L. Bischoff, S. Wickles, O. Berninghausen, E. O. van der Sluis, R. Beckmann, Visualization of a polytopic membrane protein during SecY-mediated membrane insertion. *Nat. Commun.* **5**, 4103 (2014).

24. N. Shimohata, S. Nagamori, Y. Akiyama, H. R. Kaback, K. Ito, SecY alterations that impair membrane protein folding and generate a membrane stress. *J. Cell Biol.* **176**, 307–317 (2007).

25. H. Lee, H. Kim, Membrane topology of transmembrane proteins: determinants and experimental tools. *Biochem. Biophys. Res. Commun.* **453**, 268–276 (2014).

26. R. E. Dalbey, A. Kuhn, L. Zhu, D. Kiefer, The membrane insertase YidC. *Biochim. Biophys. Acta* **1843**, 1489–1496 (2014).

27. Y. Kuruma, K. Nishiyama, Y. Shimizu, M. Muller, T. Ueda, Development of a minimal cell-free translation system for the synthesis of presecretory and integral membrane proteins. *Biotechnol. Prog.* **21**, 1243–1251 (2005).

28. I. Avcilar-Kucukgoze, H. Gamper, Y. M. Hou, A. Kashina, Purification and use of tRNA for enzymatic post-translational addition of amino acids to proteins. *STAR Protoc.* **1**, 100207 (2020).

29. M. Busch, C. R. Hernandez, M. Kamel, Y. Schaumkessel, E. van der Sluis, O. Berninghausen, T. Becker, R. Beckmann, A. Kedrov, Substrate-induced assembly and functional mechanism of the bacterial membrane protein insertase SecYEG-YidC. *bioRxiv*. 10.1101/2025.05.26.656142. (2025).

30. A. Sundaram, M. Yamsek, F. Zhong, Y. Hooda, R. S. Hegde, R. J. Keenan, Substrate-driven assembly of a translocon for multipass membrane proteins. *Nature* **611**, 167–172 (2022).

31. L. Smalinskaite, M. K. Kim, A. J. O. Lewis, R. J. Keenan, R. S. Hegde, Mechanism of an intramembrane chaperone for multipass membrane proteins. *Nature* **611**, 161–166 (2022).

32. A. Muller-Lucks, S. Bock, B. Wu, E. Beitz, Fluorescent in situ folding control for rapid optimization of cell-free membrane protein synthesis. *PLOS ONE* **7**, e42186 (2012).

33. M. L. Jacobs, M. A. Boyd, N. P. Kamat, Diblock copolymers enhance folding of a mechanosensitive membrane protein during cell-free expression. *Proc. Natl. Acad. Sci. U.S.A.* **116**, 4031–4036 (2019).

34. P. B. Wolfe, M. Rice, W. Wickner, Effects of two sec genes on protein assembly into the plasma membrane of Escherichia coli. *J. Biol. Chem.* **260**, 1836–1841 (1985).

35. E. N. Houben, C. M. ten Hagen-Jongman, J. Brunner, B. Oudega, J. Luirink, The two membrane segments of leader peptidase partition one by one into the lipid bilayer via a Sec/YidC interface. *EMBO Rep.* **5**, 970–975 (2004).

36. R. S. Osborne, T. J. Silhavy, PrlA suppressor mutations cluster in regions corresponding to three distinct topological domains. *EMBO J.* **12**, 3391–3398 (1993).

37. P. Kuhn, B. Weiche, L. Sturm, E. Sommer, F. Drepper, B. Warscheid, V. Sourjik, H. G. Koch, The bacterial SRP receptor, SecA and the ribosome use overlapping binding sites on the SecY translocon. *Traffic* **12**, 563–578 (2011).

38. K. Mitra, C. Schaffitzel, T. Shaikh, F. Tama, S. Jenni, C. L. Brooks III, N. Ban, J. Frank, Structure of the E. coli protein-conducting channel bound to a translating ribosome. *Nature* **438**, 318–324 (2005).

39. J. F. Menetret, J. Schaletzky, W. M. Clemons Jr., A. R. Osborne, S. S. Skanland, C. Denison, S. P. Gygi, D. S. Kirkpatrick, E. Park, S. J. Ludtke, T. A. Rapoport, C. W. Akey, Ribosome binding of a single copy of the SecY complex: implications for protein translocation. *Mol. Cell* **28**, 1083–1092 (2007).

40. J. W. Puziss, S. M. Strobel, P. J. Bassford Jr., Export of maltose-binding protein species with altered charge distribution surrounding the signal peptide hydrophobic core in Escherichia coli cells harboring prl suppressor mutations. *J. Bacteriol.* **174**, 92–101 (1992).

41. H. Mori, Y. Shimizu, K. Ito, Superactive SecY variants that fulfill the essential translocation function with a reduced cellular quantity. *J. Biol. Chem.* **277**, 48550–48557 (2002).

42. W. A. Prinz, C. Spiess, M. Ehrmann, C. Schierle, J. Beckwith, Targeting of signal sequenceless proteins for export in Escherichia coli with altered protein translocase. *EMBO J.* **15**, 5209–5217 (1996).

43. B. Van den Berg, W. M. Clemons Jr., I. Collinson, Y. Modis, E. Hartmann, S. C. Harrison, T. A. Rapoport, X-ray structure of a protein-conducting channel. *Nature* **427**, 36–44 (2004).

44. X. Ou, C. Ma, D. Sun, J. Xu, Y. Wang, X. Wu, D. Wang, S. Yang, N. Gao, C. Song, L. Li, SecY translocon chaperones protein folding during membrane protein insertion. *Cell* **188**, 1912–1924.e13 (2025).

45. A. Tsirigotaki, K. E. Chatzi, M. Koukaki, J. De Geyter, A. G. Portaliou, G. Orfanoudaki, M. F. Sardis, M. B. Trelle, T. J. D. Jorgensen, S. Karamanou, A. Economou, Long-Lived Folding Intermediates Predominate the Targeting-Competent Secretome. *Structure* **26**, 695–707.e5 (2018).

46. R. Freudl, Signal peptides for recombinant protein secretion in bacterial expression systems. *Microb. Cell Fact.* **17**, 52 (2018).

47. T. Pleiner, M. Bates, D. Gorlich, A toolbox of anti-mouse and anti-rabbit IgG secondary nanobodies. *J. Cell Biol.* **217**, 1143–1154 (2018).

48. E. J. Jeon, S. M. Lee, H. S. Hong, K. J. Jeong, Design of fully synthetic signal peptide library and its use for enhanced secretory production of recombinant proteins in Corynebacterium glutamicum. *Microb. Cell Fact.* **23**, 252 (2024).

49. R. Freudl, S. MacIntyre, M. Degen, U. Henning, Alterations to the signal peptide of an outer membrane protein (OmpA) of Escherichia coli K-12 can promote either the cotranslational or the posttranslational mode of processing. *J. Biol. Chem.* **263**, 344–349 (1988).

50. J. Gennity, J. Goldstein, M. Inouye, Signal peptide mutants ofEscherichia coli. *J. Bioenerg. Biomembr.* **22**, 233–269 (1990).

51. H. Mori, K. Ito, An essential amino acid residue in the protein translocation channel revealed by targeted random mutagenesis of SecY. *Proc. Natl. Acad. Sci. U.S.A.* **98**, 5128–5133 (2001).

52. J. P. van der Wolk, P. Fekkes, A. Boorsma, J. L. Huie, T. J. Silhavy, A. J. Driessen, PrlA4 prevents the rejection of signal sequence defective preproteins by stabilizing the SecA-SecY interaction during the initiation of translocation. *EMBO J.* **17**, 3631–3639 (1998).

53. G. Matsumoto, H. Nakatogawa, H. Mori, K. Ito, Genetic dissection of SecA: suppressor mutations against the secY205 translocase defect. *Genes Cells* **5**, 991–999 (2000).

54. H. M. Salis, E. A. Mirsky, C. A. Voigt, Automated design of synthetic ribosome binding sites to control protein expression. *Nat. Biotechnol.* **27**, 946–950 (2009).

55. Y. J. Chen, P. Liu, A. A. Nielsen, J. A. Brophy, K. Clancy, T. Peterson, C. A. Voigt, Characterization of 582 natural and synthetic terminators and quantification of their design constraints. *Nat. Methods* **10**, 659–664 (2013).

56. F. X. Lehr, A. Gaizauskaite, K. E. Lipinska, S. Gilles, A. Sahoo, R. Inckemann, H. Niederholtmeyer, Modular golden gate assembly of linear DNA templates for cell-free prototyping. *Methods Mol. Biol.* **2850**, 197–217 (2025).

57. B. J. Rasor, B. Vögeli, M. C. Jewett, A. S. Karim, "Cell-Free Protein Synthesis for High-Throughput Biosynthetic PathwayBiosynthetic pathways Prototyping" in *Cell-Free Gene Expression: Methods and Protocols*, A. S. Karim, M. C. Jewett, Eds. (Springer US, New York, NY, 2022), pp. 199–215.

58. A. Didovyk, T. Tonooka, L. Tsimring, J. Hasty, Rapid and scalable preparation of bacterial lysates for cell-free gene expression. *ACS Synth. Biol.* **6**, 2198–2208 (2017).

59. Z. Z. Sun, C. A. Hayes, J. Shin, F. Caschera, R. M. Murray, V. Noireaux, Protocols for implementing an Escherichia coli based TX-TL cell-free expression system for synthetic biology. *J. Vis. Exp.* , e50762 (2013).

60. A. Pandi, C. Diehl, A. Yazdizadeh Kharrazi, S. A. Scholz, E. Bobkova, L. Faure, M. Nattermann, D. Adam, N. Chapin, Y. Foroughijabbari, C. Moritz, N. Paczia, N. S. Cortina, J. L. Faulon, T. J. Erb, A versatile active learning workflow for optimization of genetic and metabolic networks. *Nat. Commun.* **13**, 3876 (2022).

61. S. Barthel, M. Hoffmann-Becking, I. G. Karimov, T. J. Erb, Genetically Encoded Control of In Vitro Transcription-Translation Coupled DNA Replication. *ACS Synth. Biol.* **14**, 4134–4142 (2025).

62. C. R. Zito, E. Antony, J. F. Hunt, D. B. Oliver, M. M. Hingorani, Role of a conserved glutamate residue in the Escherichia coli SecA ATPase mechanism. *J. Biol. Chem.* **280**, 14611–14619 (2005).

63. A. Robson, A. E. Booth, V. A. Gold, A. R. Clarke, I. Collinson, A large conformational change couples the ATP binding site of SecA to the SecY protein channel. *J. Mol. Biol.* **374**, 965–976 (2007).

64. S. Kavousipour, S. Mohammadi, E. Eftekhar, M. Barazesh, M. H. Morowvat, In silico investigation of signal peptide sequences to enhance secretion of CD44 nanobodies expressed in escherichia coli. *Curr. Pharm. Biotechnol.* **22**, 1192–1205 (2021).
